# LSD degrades hippocampal spatial representations and suppresses hippocampal-visual cortical interactions

**DOI:** 10.1016/j.celrep.2021.109714

**Published:** 2021-09-14

**Authors:** Carli Domenico, Daniel Haggerty, Xiang Mou, Daoyun Ji

**Affiliations:** 1 Department of Neuroscience, Baylor College of Medicine, Houston, TX 77030, USA; 2 Department of Molecular and Cellular Biology, Baylor College of Medicine, Houston, TX 77030, USA; 3 These authors contributed equally; 4 Lead contact

## Abstract

Lysergic acid diethylamide (LSD) produces hallucinations, which are perceptions uncoupled from the external environment. How LSD alters neuronal activities *in vivo* that underlie abnormal perceptions is unknown. Here, we show that when rats run along a familiar track, hippocampal place cells under LSD reduce their firing rates, their directionality, and their interaction with visual cortical neurons. However, both hippocampal and visual cortical neurons temporarily increase firing rates during head-twitching, a behavioral signature of a hallucination-like state in rodents. When rats are immobile on the track, LSD enhances cortical firing synchrony in a state similar to the wakefulness-to-sleep transition, during which the hippocampal-cortical interaction remains dampened while hippocampal awake reactivation is maintained. Our results suggest that LSD suppresses hippocampal-cortical interactions during active behavior and during immobility, leading to internal hippocampal representations that are degraded and isolated from external sensory input. These effects may contribute to LSD-produced abnormal perceptions.

## In brief

Domenico et al. investigate how lysergic acid diethylamide (LSD) affects hippocampal place cells and visual cortical neurons. During active navigation, LSD reduces hippocampal-visual cortical correlation and hippocampal spatial representation precision. During immobile behavior, LSD promotes isolated hippocampal reactivation and produces a cortical state similar to wakefulness-to-sleep transition.

## Graphical abstract

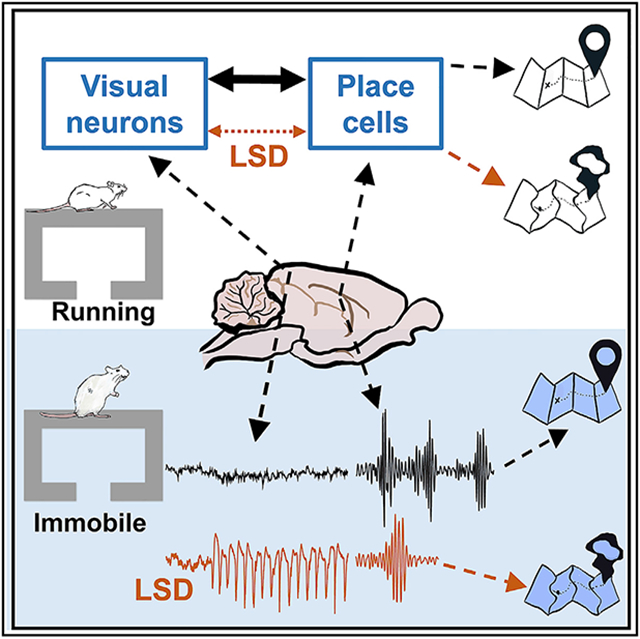

## INTRODUCTION

The psychedelic drug lysergic acid diethylamide (LSD) is a potent hallucinogen that produces surreal hallucinations in humans, defined as subjective perceptions uncoupled from external environments (Liechti, 2017; Schmid et al., 2014). Previous studies in humans suggest that LSD and similar hallucinogens disrupt activities in the prefrontal cortex, visual cortex (VC), and the hippocampus (HP), as well as their functional connections with other regions, during resting (Alamia et al., 2020; Carhart-Harris et al., 2012, 2016; Müller et al., 2018; Palhano-Fontes et al., 2015; Preller et al., 2019; Roseman et al., 2014; Tagliazucchi et al., 2014, 2016) and during active tasks (Schmidt et al., 2018). However, how LSD alters firing activities of neurons in these areas *in vivo* is unknown.

Unlike human studies, neuronal firing activity in animals can be recorded *in vivo* during behavioral responses to psychedelic drugs. We set out to study the effects of LSD on neuronal activities in freely moving rats. Previous behavioral studies in rodents identified a number of behavioral changes, including reduced movement with increased immobility (but sometimes enhanced mobility possibly depending on dosage and timing) (Adams and Geyer, 1982; Mittman and Geyer, 1991; Pálenícek et al., 2010). In addition, rodents display a unique response to LSD and similar hallucinogens called head-twitching (HT), which is a brief, rapid shake of the head in rats and mice (Corne et al., 1963; Fantegrossi et al., 2008; González-Maeso et al., 2007) or a head-bobbing motion in rabbits (Dave et al., 2004; Romano et al., 2010). HT is considered a behavioral signature of the LSD-produced mental state similar to human hallucination (Fantegrossi et al., 2008). HT in rodents and altered perceptions in humans caused by hallucinogens like LSD require activation of the serotonin 5-hydroxytryptamine-2-A receptor (5HT_2A_R), which can be blocked by specific 5HT_2A_R antagonists such as M100907 (Fantegrossi et al., 2008; González-Maeso et al., 2007; Schreiber et al., 1995). Previous studies show that the LSD-induced HT depends on HP (Dave et al., 2004; Romano et al., 2010). Furthermore, HP, as well as VC, is also important for visual hallucinations (Carhart-Harris et al., 2016; Vignal et al., 2007). In this study, we targeted neuronal firing activities, as well as local field potentials (LFPs), in the CA1 area of HP and VC in freely moving rats during their behavioral responses to LSD.

HP place cells fire spikes at one or a few places (place fields) of an environment (O’Keefe and Dostrovsky, 1971). A population of place cells with place fields covering an environment is believed to encode an internal cognitive map of the environment (O’Keefe and Nadel, 1978; Wilson and McNaughton, 1993). Place fields are formed by integrating self-motion cues with external sensory input, especially visual cues (Knierim and Hamilton, 2011; Moser et al., 2017; Muller, 1996). Indeed, during active maze running when CA1 LFPs display prominent theta (6–10 Hz) oscillations (Buzsáki, 2002), the firing activities of CA1 and VC neurons are correlated (Haggerty and Ji, 2015). During resting and immobile behavior, firing activity patterns in CA1 are reactivated, possibly for planning or memory recall, at times when CA1 LFPs display high-frequency (100–250 Hz) ripple oscillations (Carr et al., 2011; Foster and Wilson, 2006; Gupta et al., 2010; Karlsson and Frank, 2009; Pfeiffer and Foster, 2013; Wilson and McNaughton, 1994; Wu et al., 2017). In addition, CA1 and VC are coordinately reactivated during sleep for memory consolidation (Ji and Wilson, 2007). Therefore, CA1 and VC are engaged in functional interactions in various behavioral states.

Because LSD produces a mismatch between internal perception and external environment, we aimed to study whether the cognitive map in HP and the interaction between HP and VC are altered by LSD *in vivo.* To this end, we recorded firing activities of CA1 and VC neurons and LFPs in rats while they ran a familiar track before and after systemic injection of LSD. We analyzed whether and how LSD altered CA1 and VC neuronal activities during active running and during immobility on the track, focusing on place-coding properties of CA1 place cells and their interactions with VC neurons.

## RESULTS

**Figure 1.**
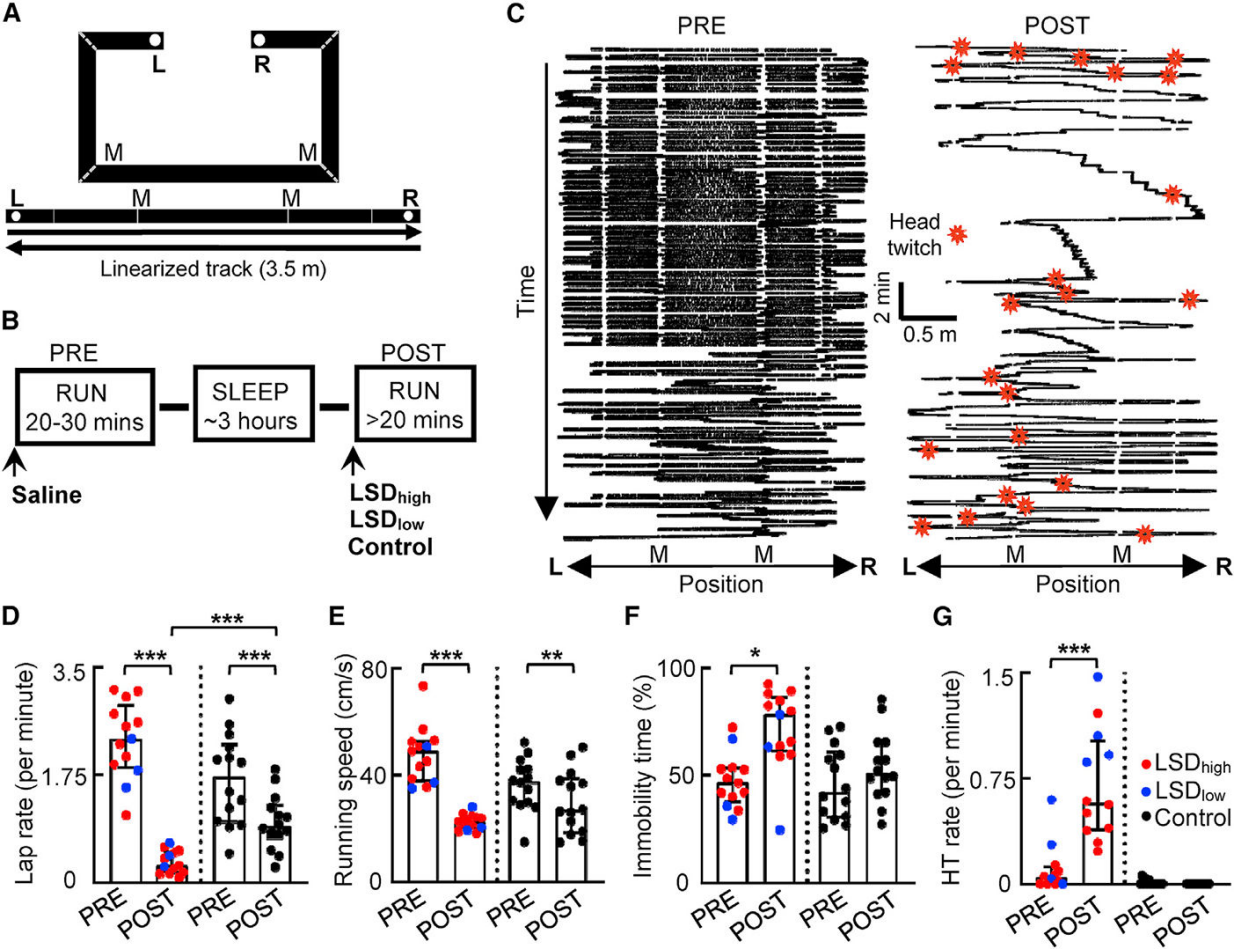
LSD enhanced head twitching and immobility (A) C-shaped track (top) and the linearized trajectories (bottom). L and R, reward locations; M, landmark locations (two corners of the long arm). (B) Behavioral procedure is as follows: two track-running sessions (PRE and POST), with each following a drug administration, separated by a sleep session. Prior to PRE, saline was injected. Prior to POST, either LSD_high_, LSD_low_, or a control (Ctrl) condition (injection of the 5HT_2A_R antagonist M100907 followed by injection of LSD_low_, M100907 alone, or saline alone) was administered. (C) Linearized spatial trajectories (black lines) and head twitches (HTs; red stars) of an example rat under LSD_high_ in PRE and POST. White gaps, linearization artifacts when the animal made sharp turns at corners. M, two of the corners. (D–G) Lap rate (D), running speed (E), percentage of immobile time (F), and HT rate (G) in PRE and POST under LSD_high_ (n = 10), LSD_low_ (n = 3, except for G, which is n = 4), and the Ctrl (n = 14) conditions. Each dot is a session. Boxplot: median and [25% 75%] range values; same in other figures. *p < 0.05, **p < 0.01, ***p < 0.001.

We trained rats to run laps back and forth (two trajectories) on a familiar C-shaped track (Figure 1A) prior to the recording experiment. On each recording day, rats performed the same running task for two sessions (PRE and POST), separated by a sleep session (Figure 1B). Fifteen minutes prior to PRE, rats received an injection of the saline vehicle. Fifteen minutes prior to POST, rats received another drug administration under various conditions (Figure 1B), namely, injection of LSD at either a high (LSD_high_) or low (LSD_low_) dose or one of the control injections including the 5HT_2A_R antagonist M100907, followed by LSD_low_, antagonist alone, or saline alone. We recorded 781 CA1 neurons and 153 VC cells from 17 rats on 20 recording days, with most of the rats (n = 14) only recorded 1 day per animal (Table S1; see STAR Methods). Six additional rats were used only for the behavioral experiment and/or LFP recordings without single-neuron activities, following the same track running and injection schedule.

### Reduced running and increased HT

To examine behavioral responses to LSD in our track-running task, we linearized the two trajectories on the track (Figure 1C) and quantified running behavior by the number of laps per minute (lap rate), running speed, and percentage of immobility time in each session. For the quantification of immobility, because the duration of POST varied from animal to animal and was longer than that of PRE, which could naturally lead to more time in immobility, we limited the quantification to the first 30 min of POST, a duration comparable to PRE.

The median lap rate in POST under LSD (0.27 [0.16, 0.50], median [25%, 75%] values, same below unless otherwise specified) was dramatically reduced (88% lower) from PRE (2.3 [1.9, 2.9]; p = 2.4 × 10^−4^, Wilcoxon rank-sum test), whereas the reduction was modest (46% lower) under the control (PRE: 1.7 [0.98, 2.2], POST: 0.91 [0.72, 1.3], p = 1.2 × 10^−4^) condition (Figure 1D). Although both groups demonstrated reduced laps in POST, the lap rate in POST under LSD was significantly lower than that under the control (p = 5.1 × 10^−5^, Mann-Whitney test) condition. The median running speed (after removing immobility periods) under LSD was also significantly reduced (54% lower) in POST from PRE (PRE: 48 [40, 53] cm/s, POST: 22 [20, 53] cm/s, p = 1.4 × 10^−10^, Wilcoxon rank-sum test) and modestly reduced (29% lower) under the control (PRE: 38 [30, 43] cm/s, POST: 27 [19, 39] cm/s, p = 0.0067) condition (Figure 1E). Accordingly, the median percentage of immobile time under LSD in the first 30 min of POST was significantly increased (34% higher) from PRE (PRE: 40% [38%, 53%], POST: 78% [61%, 86%], p = 0.017, Wilcoxon rank-sum test) but not under the control (PRE: 42% [31%, 61%]; POST: 51% [42%, 65%], p = 0.27) condition (Figure 1F). The data suggest that the rats ran fewer laps and ran slower in POST than in PRE, but this change was greater under LSD than the control condition. Furthermore, the animals spent more time immobile in POST under LSD but not under the control condition, consistent with previous studies (Mittman and Geyer, 1991; Pálenícek et al., 2010).

We then analyzed the HT events, which were identified from those animals video recorded on the track. HT rarely occurred in PRE but frequently occurred in POST under LSD across all locations of running trajectories (Figure 1C). The HT rate (number of HTs per minute) in POST under LSD was significantly higher than that in PRE (PRE: 0.047 [0.0, 0.12], POST: 0.58 [0.38, 1.0]; p = 4.9 × 10^−4^, Wilcoxon rank-sum test; Figure 1G). No increase in HT was observed in POST under the control condition (PRE: 0.0 [0.0, 0.031], POST: 0.0 [0.0, 0.0], p = 0.25). The HT rate was even higher in POST under LSD_low_ (0.98 [0.88, 1.4], n = 4 sessions) than that under LSD_high_ (0.45 [0.31, 0.58], n = 8, p = 0.028, Mann-Whitney test). When LSD was injected at the same low dose after the injection of M100907, HT was abolished in POST (PRE: 0.0 [0.0, 0.038], POST: 0.0 [0.0, 0.0], n = 6, p = 0.50). This result shows that LSD worked as expected from previous reports, inducing 5HT_2A_R-dependent HTs (González-Maeso et al., 2007; Schreiber et al., 1995).

### Reduced firing rates of CA1 and VC neurons

We asked how firing activities of CA1 and VC cells were altered by LSD. We first analyzed the CA1 and VC cells that were active (firing rate, >0.5 Hz) on at least one of the trajectories in PRE or POST (active cells). A total of 365 active CA1 and 130 active VC cells were identified (Table S1). We examined the overall firing rates of these neurons during active running. The firing rates of CA1 cells were lower under LSD in POST (Figure S1A). The median rate was reduced from PRE to POST under LSD_high_ (PRE: 1.3 [0.67, 2.0] Hz, POST: 0.45 [0.054, 0.89] Hz; p = 9.8 × 10^−22^, Wilcoxon rank-sum test) and under LSD_low_ (PRE: 1.2 [0.64, 2.1] Hz, POST: 0.92 [0.58, 1.6] Hz; p = 0.015) but not under the control (PRE: 1.1 [0.59, 2.5] Hz, POST: 1.3 [0.62, 2.2] Hz; p = 0.57) condition. However, the firing rates of VC neurons were not altered by LSD (Figure S1A). Their median rate was not significantly different between PRE and POST under LSD_high_ (PRE: 3.3 [1.5, 8.7] Hz, POST: 3.1 [1.1, 6.9] Hz; p = 0.22), LSD_low_ (PRE: 5.0 [2.5, 10] Hz, POST: 6.1 [0.28, 8.8] Hz; p = 0.63), or the control (PRE: 4.0 [1.7, 8.1] Hz, POST: 3.7 [1.2, 8.9] Hz; p = 0.063) condition.

A question is whether the firing rate changes in CA1 resulted primarily from the speed changes between PRE and POST. To address this question, we removed the effect of speed by using a regression analysis (see STAR Methods) and computed the average residual rate for PRE and POST (Figure S1B). We found that the average residual rates of CA1 neurons remained significantly lower in POST than in PRE under LSD_high_ (PRE: 0.053 [0.0053, 0.10] Hz, POST: −0.43 [−1.0, −0.047] Hz; p = 3.1 × 10^−15^, Wilcoxon rank-sum test) and LSD_low_ (PRE: 0.033 [−0.059, 0.11] Hz, POST: −0.20 [−0.58, 0.18] Hz; p = 0.024) but not under the control (PRE: −0.026 [−0.20, 0.21] Hz, POST: 0.11 [−0.43, 0.42] Hz; p = 0.34) condition (Figure S1C). The average residual rates of VC cells were not significantly different between POST and PRE under LSD_high_ (PRE: 0.023 [−0.10, 0.23] Hz, POST: −0.18 [−1.6, 0.43] Hz; p = 0.55), LSD_low_ (PRE: 0.017 [−0.31, 0.66] Hz, POST: −0.52 [−1.5, 0.60] Hz; p = 0.81), or the control (PRE: −0.14 [−0.53, 0.079] Hz, POST: 0.26 [−0.49, 2.2] Hz; p = 0.25) condition (Figure S1C). Thus, the effects of LSD on CA1 cell firing rates during running remained even after removing the speed modulation.

We also examined firing rates of active CA1 and VC cells during immobility. The median rate of CA1 cells was significantly lower in POST than PRE under LSD_high_ (PRE: 0.21 [0.073, 0.53] Hz, POST: 0.073 [0.023, 0.24] Hz; p = 6.1 × 10^−14^, Wilcoxon rank-sum test) but not under LSD_low_ (PRE: 0.27 [0.11, 0.61] Hz, POST: 0.25 [0.076, 0.49] Hz; p = 0.45) or the control (PRE: 0.21 [0.063, 0.50] Hz, POST: 0.21 [0.071, 0.62] Hz; p = 0.73) condition (Figure S1D). Similarly, the median rate of VC cells in POST was significantly reduced from that of PRE under LSD_high_ (PRE: 3.1 [1.0, 7.0] Hz, POST: 2.2 [0.70, 5.3] Hz; p = 0.0097) but not under LSD_low_ (PRE: 5.2 [2.9, 8.8] Hz, POST: 4.7 [0.35, 8.9] Hz; p = 0.82) or the control (PRE: 3.7 [1.1, 7.6] Hz, POST: 3.0 [0.79, 8.0] Hz; p = 0.36) condition (Figure S1D).

Taken together, LSD reduced the firing rates of active CA1 and VC cells in a behavior- and dose-dependent manner. LSD_low_ had a relatively moderate effect, reducing the rates of CA1 active cells during running. LSD_high_ had a broader effect, reducing rates during both running and immobility and also reducing rates of active VC cells during immobility. In addition, we found that the firing rates of putative CA1 interneurons (firing rate >5 Hz) and those CA1 cells that were inactive (firing rate <0.5 Hz) during running (silent cells) were also reduced by LSD during running (Figure S1E) and during immobility (Figure S1F), suggesting that LSD appeared to affect all cell types in CA1.

### Increased firing rates around HT

**Figure 2.**
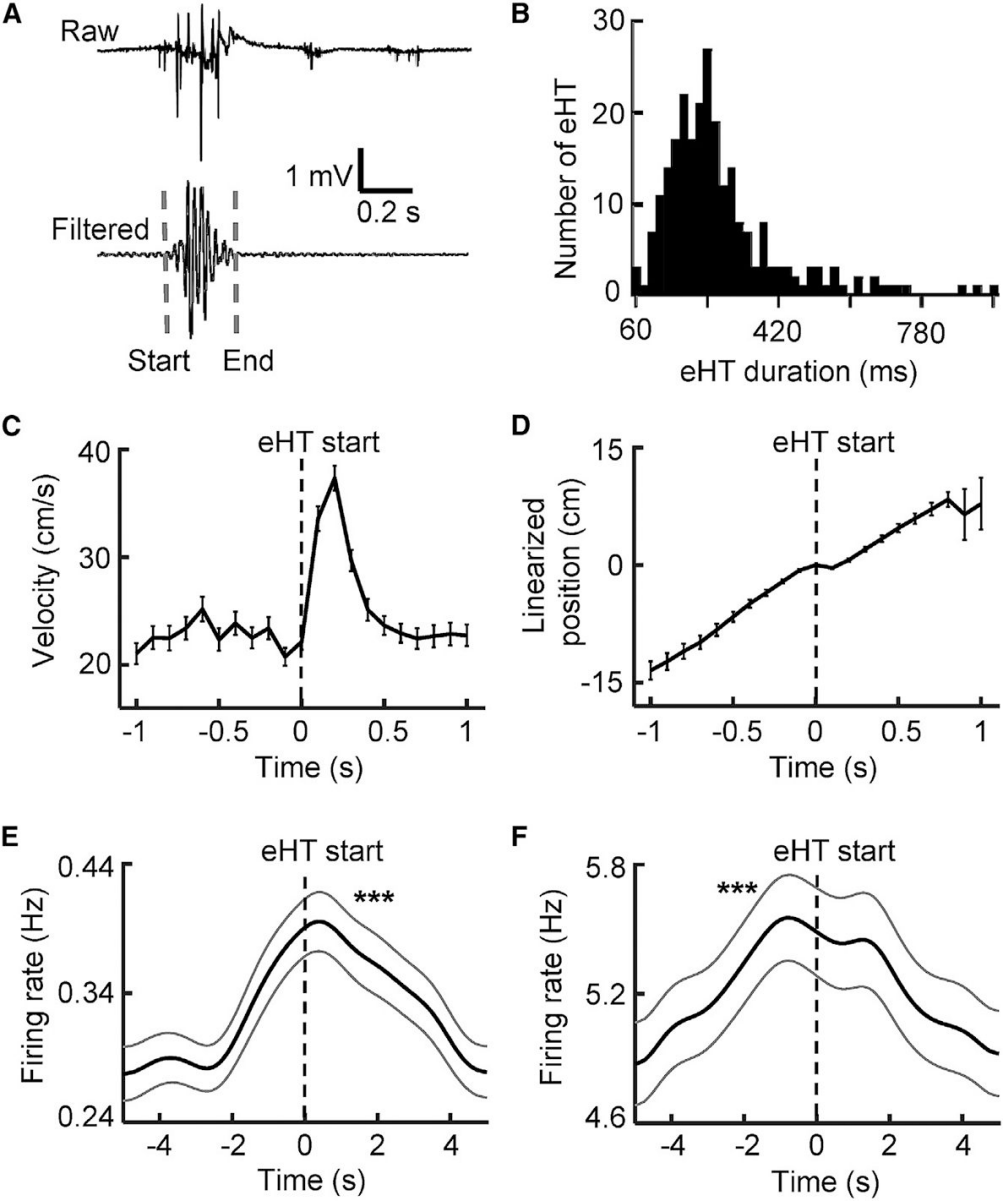
Increased firing rates of CA1 and VC cells around head twitching (A) An example HT event identified by EMG (eHT). Raw, raw EMG trace; filtered, the same EMG trace filtered within 20–60 Hz; dashed line, eHT start/end time. (B) Distribution of eHT duration (n = 245 eHTs). (C) Average velocity (mean ± SE) of head movement triggered by eHT start times (time 0). (D) Average linearized position (mean ± SE) along the moving trajectory relative to the eHT start times. The linearized positions at eHT start times were aligned to position 0. (E and F) Average firing rate (mean ± SE) of active CA1 (E) and VC (F) cells triggered by the eHT start times (n = 218 eHTs). ***p < 0.001 for peak value. See also Figure S1.

Because HT is considered a behavioral signature of 5HT_2A_R-mediated hallucinations (Fantegrossi et al., 2008), we examined the behavior and neural activities surrounding HTs. We took advantage of the electromyography (EMG) signals recorded from the neck muscle in a group of rats under LSD_high_ and LSD_low_ (N = 8, Table S1). EMGs displayed a large deflection whenever an HT event occurred, which allowed us to precisely determine its start and end times (Figure 2A). Because HTs were rare in PRE and in control rats, we analyzed only the EMG-detected HT events (eHTs) in POST under LSD (LSD_high_ and LSD_low_ combined). The median duration of eHTs was 236 [176, 306] ms (Figure 2B).

As expected, the velocity of head movement increased immediately at eHT start times and lasted slightly after eHT end times for a total duration of ~300 ms (Figure 2C). The animals’ movement along the one-dimensional linearized trajectory slowed down between 100 ms before and 100 ms after eHT start times but resumed afterward (Figure 2D). The analysis indicates that HTs occurred mostly when rats were running along a trajectory, slowed, head-shook quickly, and then continued running.

To understand whether neural activities were altered during eHTs, we computed the average firing rates of active CA1 and VC cells triggered by eHT start times. We found that both CA1 and VC cells significantly increased their rates around eHTs (CA1: p = 5.0 × 10^−113^; VC: p = 1.0 × 10^−116^, one-way ANOVA; Figures 2E and 2F). The increase appeared to be broad (>4 s), starting before the eHT start times and ending well beyond the eHT end times. In addition, it appeared that VC rates peaked 0.8 s before the eHT start times, but CA1 rates peaked 0.4 s after the eHT start times, suggesting that the VC changes led the CA1’s changes. Therefore, a temporary, broad firing rate increase occurred before and during HTs, despite an overall rate reduction in POST under LSD.

### Altered spatial properties of CA1 place cells

**Figure 3.**
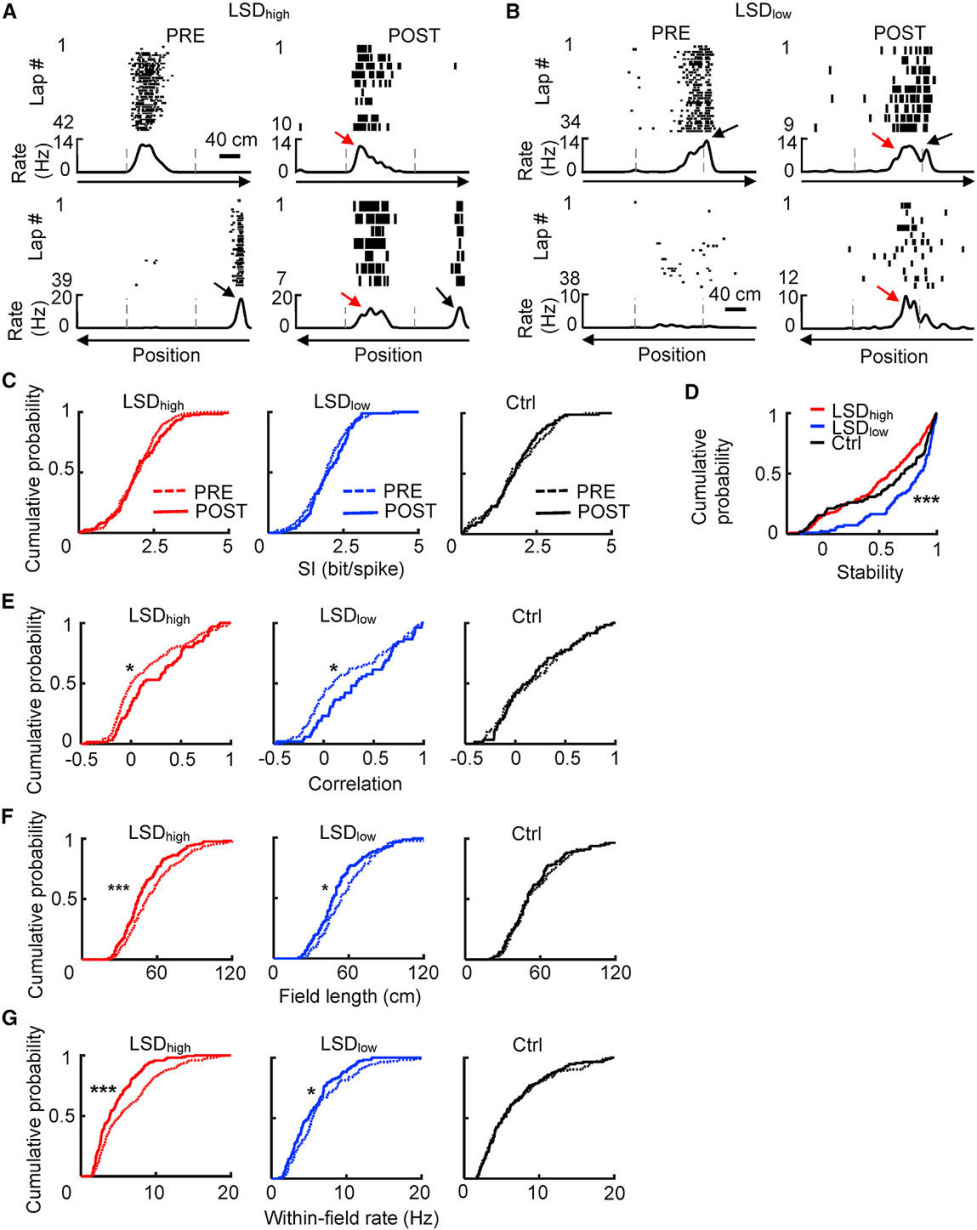
CA1 place cells under LSD maintained similar firing locations but had reduced directionality and within-field firing rates (A and B) Example place cells under LSD_high_ (A) and under LSD_low_ (B). Each panel shows the lap-by-lap spike raster (top) of a cell and its rate curves (bottom) during running on the two opposite, linearized trajectories of the C-track in PRE and POST. Dashed lines, landmark positions on the track; black arrows, dominant place field in PRE had similar firing locations with reduced rates in POST; red arrows, place field became more bi-directional in POST. (C) Cumulative distributions of spatial information (SI) under LSD_high_ (PRE: n = 244 cell × trajectories, POST: n = 121), LSD_low_ (PRE: n = 101, POST: n = 83), and the Ctrl conditions (PRE: n = 85, POST: n = 84) for place cells on active trajectories in PRE and POST. (D) Cumulative distributions of stability under LSD_high_ (n = 204 cell × trajectories), LSD_low_ (n = 98), and the Ctrl (n = 104) condition. (E) Cumulative distributions of correlation between rate curves of two opposite trajectories, as a measure of directionality under LSD_high_ (PRE: n = 175 cells, POST: n = 66), LSD_low_ (PRE: n = 72, POST: n = 52), and the Ctrl conditions (PRE: n = 58, POST: n = 55). (F and G) Same as (E), but for field length (F) and within-field firing rate (G) under LSD_high_ (PRE: n = 286 fields, POST: n = 167), LSD_low_ (PRE: n = 117, POST: n = 112), and the Ctrl (PRE: n = 114, POST: n = 113) conditions. *p < 0.05, ***p < 0.001. See also Figures S1-S4.

Given the firing rate changes during running, we asked how the place-coding properties of CA1 place cells were affected by LSD. We plotted a spike raster of example place cells and their firing rates at positions along a linearized trajectory (rate curve) in PRE and POST under LSD_high_ and LSD_low_ (Figures 3A and 3B; Figure S2). The plots suggest that place field locations were relatively preserved between PRE and POST, but firing rates within place fields were reduced, especially under LSD_high_. In addition, place cells appeared more likely to fire at the same locations along the two opposite trajectories (directions), i.e., the directionality of place fields was reduced under LSD (Figures 3A and 3B; Figure S2). We used several measures to quantify these observations, starting with overall properties of firing rate curves, followed by properties of individual place fields. These measures were computed from firing activities during active running, excluding those occurring during immobility and at both ends of the track.

First, the spatial tuning of a place cell on an active trajectory was quantified by spatial information (SI; Figure 3C). The median SI values were not significantly different between PRE and POST under LSD_high_ (PRE: 2.0 [1.4, 2.4] bits/spike; POST: 1.9 [1.4, 2.6] bits/spike, n = 121; p = 0.46, Mann-Whitney test), LSD_low_ (PRE: 1.9 [1.4, 2.4] bits/spike; POST: 2.0 [1.5, 2.6] bits/spike; p = 0.33), or the control (PRE: 1.6 [1.2, 2.5] bits/spike; POST: 1.8 [1.2, 2.3] bits/spike; p = 0.81) condition.

Second, we quantified the stability of place cell firing locations on active trajectories between PRE and POST (Figure 3D). The median stability values were significantly different among LSD_high_ (0.59 [0.21, 0.85]), LSD_low_ (0.85 [0.61, 0.94]), and the control (0.71 [0.22, 0.91]) condition (p = 5.6 × 10^−7^, Kruskal-Wallis test). Post hoc comparisons indicated that this difference was due to the higher median stability of LSD_low_, relative to that of LSD_high_ (p = 5.3 × 10^−8^, Mann-Whitney test) and to that of the control (p = 0.0021), with no difference between LSD_high_ and the control (p = 0.10). The reason for the seemingly higher stability under LSD_low_ is unclear; it could be due to other factors such as the amount of experience on the familiar track (although rats were all trained on the track for >4 days). Nevertheless, our data show that LSD did not reduce the stability of place field locations.

Third, to quantify place cell firing directionality, we computed a spatial correlation between a cell’s rate curves on the two opposite trajectories in a session (Figure 3E). A higher correlation means lower directionality. We found that the median correlation value was significantly increased from PRE to POST under both LSD_high_ (PRE: 0.0 [−0.12, 0.34]; POST: 0.12 [−0.034, 0.50]; p = 0.017, Mann-Whitney test) and LSD_low_ (PRE: 0.096 [−0.097, 0.59]; POST: 0.33 [0.053, 0.67]; p = 0.049) but not under the control condition (PRE: 0.15 [−0.12, 0.43]; POST: 0.11 [−0.098, 0.45]; p = 0.95). Thus, LSD reduced the directionality of CA1 place cells.

Fourth, we detected individual place fields on each active trajectory and quantified their changes from PRE to POST by the number of place fields per trajectory, field length, and within-field firing rate. The number of fields was similar between PRE and POST under LSD_high_ [PRE: 1.1 ± 0.04 (mean ± SE), n = 244 cell × trajectories; POST: 1.1 ± 0.6, n = 133; p = 0.80, Student’s t test), LSD_low_ (PRE: 1.1 ± 0.06, n = 101; POST: 0.98 ± 0.08, n = 98; p = 0.30), and the control (PRE: 1.2 ± 0.08, n = 85; POST: 1.2 ± 0.09, n = 87; p = 0.74) condition. The median field length (Figure 3F) was slightly, but significantly, decreased from PRE to POST under LSD_high_ (PRE: 52 [40, 69] cm; POST: 45 [36, 60] cm; p = 7.2 × 10^−4^, Mann-Whitney test) and LSD_low_ (PRE: 54 [42, 71] cm; POST: 48 [38, 60] cm; p = 0.023) but not under the control (PRE: 50 [39, 71] cm; POST: 50 [39, 64] cm; p = 0.55) condition. The median within-field firing rate (Figure 3G) was significantly lower in POST than in PRE under both LSD_high_ (PRE: 5.3 [3.0, 8.9] Hz; POST: 3.8 [2.5, 6.5] Hz; p = 4.7 × 10^−6^, Mann-Whitney test) and LSD_low_ (PRE: 5.6 [3.5, 8.9] Hz; POST: 4.9 [3.0, 7.0] Hz; p = 0.029) but not under the control (PRE: 5.2 [3.1, 8.9] Hz; POST: 5.2 [3.3, 8.5] Hz; p = 0.91) condition. To address the issue that the lower within-field rate might be due to slower speed under LSD, we removed the effect of speed on within-field rates in PRE and POST. We found that the median residual within-field rate in POST remained significantly lower under LSD_high_ (PRE: 0.19 [0.058, 0.42] Hz, POST: −2.1 [−4.0, −0.76] Hz, n = 161 fields; p = 3.2 × 10^−21^, Wilcoxon rank-sum test) and LSD_low_ (PRE: 0.14 [−0.099 0.34] Hz, POST: −0.87 [−2.5, 0.26] Hz, n = 69; p = 6.4 × 10^−4^) but remained similar under the control (PRE: 0.090 [−0.92, 0.77] Hz, POST: −0.28 [−1.9, 2.2] Hz, n = 61; p = 0.80) condition (Figure S1C).

Taken together, these results indicate that place cell firing activities were largely stable under LSD, with similar SI and no reduction in stability. However, the spatial tuning became significantly less precise, with reduced directionality and a lower within-field firing rate.

Previous studies show that place cells at different locations of CA1, either in superficial versus deep layers of the pyramidal layer (Mizuseki et al., 2011) or in proximal versus distal ends of CA1 (Oliva et al., 2016; Soltesz and Losonczy, 2018), differ in their firing rates or bursting properties. We asked whether LSD differentially affected place cells with these different characteristics. First, we separated the place cells recorded under LSD into three groups, as follows: those with stable fields between PRE and POST, those with unstable fields, and those active in PRE but silent in POST. The firing rates of the POST-silent group were lower in PRE to begin with than those of the other two groups, whereas the burst index was not significantly different among the groups (Figure S3A). This result is consistent with a broad rate reduction in all place cells. Second, we divided those cells that fired on both running trajectories in PRE and POST under LSD into two groups, namely, those with reduced directionality and those with directionality unchanged or increased. We found no difference in either firing rate or burst index between the two groups (Figure S3B). In addition, we examined the identified recording sites in CA1 for rats under LSD_high_, LSD_low_, and the control condition and found they were comparable (Figure S3C). Therefore, our results suggest that LSD affected place cells broadly in CA1, consistent with the rate change of CA1 silent cells and interneurons during running (Figure S1E).

Because some drugs such as cannabinoids can affect LFPs and fine spike timing of place cells (Robbe and Buzsáki, 2009; Robbe et al., 2006), we examined how LSD affected theta oscillations in the CA1 LFPs, as well as theta phase tuning and phase precession of place cells. LSD appeared to lower theta peak frequencies, but not theta power, when compared at similar speed ranges between PRE and POST (Figures S4A and S4B). But, the coherence in the theta band between CA1 and VC LFPs was unaltered (Figure S4C). The mean theta phase and phase variance (a measure of the degree of theta phase tuning) of active cells were not significantly affected by LSD (Figures S4D and S4E). Theta phase precession within CA1 place fields was apparently intact under LSD (Figures S4F-S4H). In addition, theta sequences, measured by the pairwise correlation between place field distances and firing timing intervals within theta cycles (Dragoi and Buzsáki, 2006; Robbe and Buzsáki, 2009), appeared normal under LSD (Figure S4I). Therefore, theta oscillations were modestly affected, but theta phase precession and theta sequences of place cells remained under LSD.

### Changes in spatial representation of place cell ensembles

After analyzing individual place cells, we examined how spatial representations of CA1 place cell populations were altered by LSD. We already showed that firing rates of CA1 cells reduced with LSD, necessarily leading to fewer active cells during trajectory running in POST. Here, we focused on those locations with a sufficient number of active place cells (≥5) and asked whether the remaining active cells in POST encoded the locations similarly as in PRE at the population level. We constructed population vectors (PVs) from the active cells’ rate curves and computed a correlation between PVs at the same location of the same trajectory between PRE and POST (cross-session PV_corr_) or between PVs at the same location in the same session but between two opposite trajectories (cross-trajectory PV_corr_).

**Figure 4.**
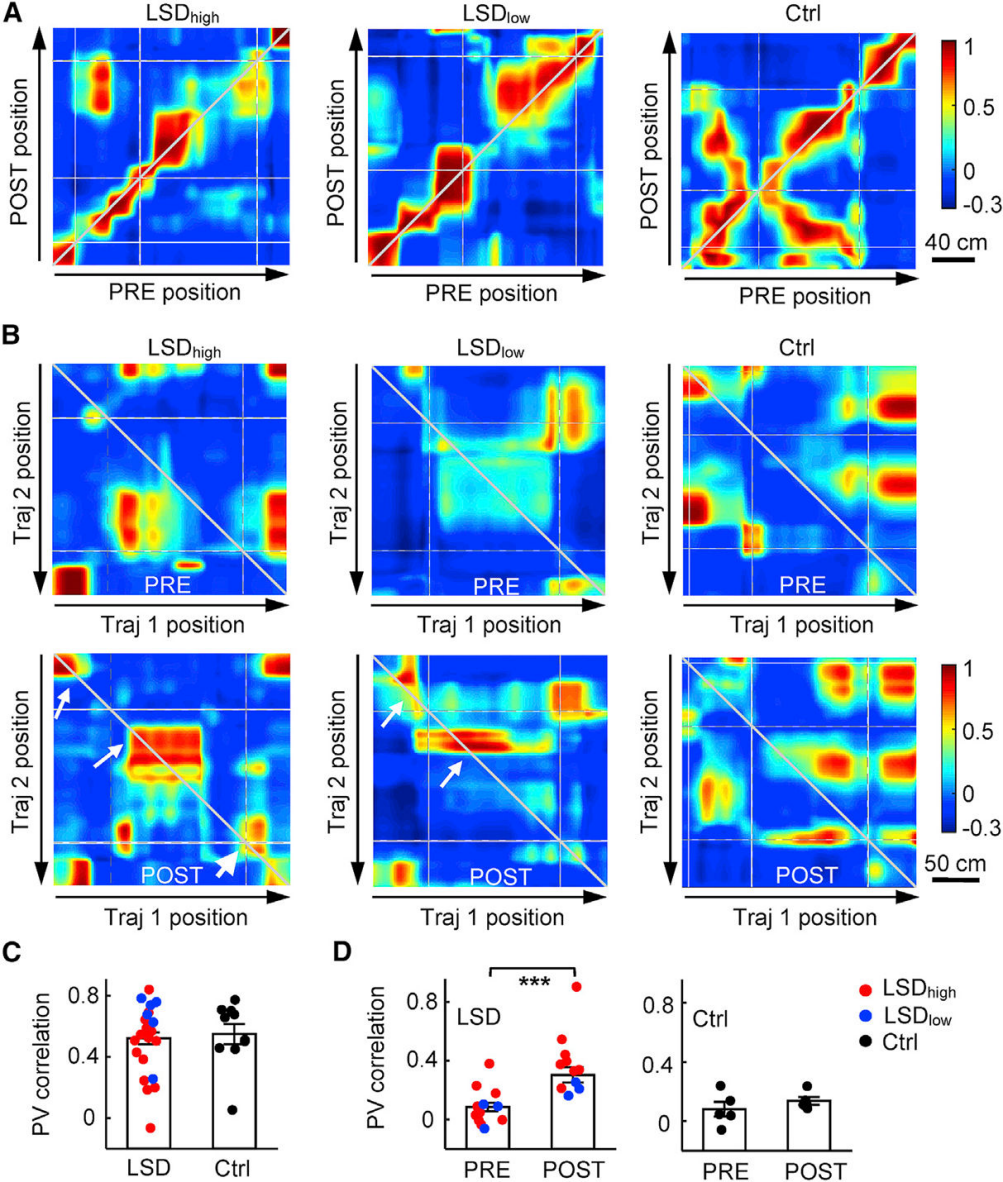
Stable representation but reduced directionality in active place cell ensembles (A) Example PV correlations of CA1 place cells between PRE and POST along the same linearized trajectory (cross-session PV_corr_) under the LSD_high_, LSD_low_, or the Ctrl condition. Arrows, running directions; gray broken lines, landmark locations; white lines, linearization artifacts when animals made sharp turns around some corners of the track. Note the similar values (colors) along the diagonal line under different conditions. (B) Same as (A), but for example PV correlations between two opposite trajectories (Traj 1 and Traj 2) in the same PRE or POST session (cross-trajectory PV_corr_). Note that values along the reverse diagonal line were higher in POST (arrows) than those in PRE under LSD_high_ and LSD_low_. (C) Average (mean ± SEM) cross-session PV_corr_ for all recording days and all animals under LSD (LSD_high_ and LSD_low_ combined, n = 22 trajectories) and the Ctrl condition (n = 10). Each dot is a trajectory. (D) Same as in (C), but for average cross-trajectory PV_corr_ in PRE and POST for LSD (PRE: n = 17 sessions, POST: n = 16) and Ctrl (PRE: n = 5, POST: n = 5). Each dot is a session. ***p < 0.001.

The cross-session PV_corr_ quantifies how stable PVs were between PRE and POST. Plots of example trajectories suggest similar PV_corr_ at the same locations under all conditions (Figure 4A). We computed an average PV_corr_ across all locations of a trajectory and compared the average PV_corr_ for all trajectories between different conditions. We found that the cross-session PV_corr_ did not significantly differ between LSD (LSD_high_ and LSD_low_ combined) and control conditions (LSD: 0.52 ± 0.04; control: 0.55 ± 0.07; p = 0.72, Student’s t-test; Figure 4C). By breaking down the LSD group to LSD_high_ and LSD_low_, we showed that the cross-session PV_corr_ under LSD_low_ was modestly higher than that under LSD_high_, but it did not reach significance (LSD_high_: 0.46 ± 0.06, n = 16 trajectories; LSD_low_: 0.64 ± 0.08, n = 6; p = 0.099). This result suggests that population-level spatial representations were largely stable under LSD for the active cells in POST, consistent with the result of stable individual place cells (Figure 3D).

The cross-trajectory PV_corr_ quantifies how similar PVs on one trajectory were to its opposite trajectory in the same session. Higher cross-trajectory PV_corr_ means lower directionality at the population level. Plots of example trajectories suggest higher cross-trajectory PV_corr_ at the same locations in POST than that in PRE under LSD_high_ and LSD_low_ but not the control condition (Figure 4B). There was a significant increase in the average cross-trajectory PV_corr_ from PRE to POST under LSD (PRE: 0.084 ± 0.029; POST: 0.30 ± 0.05; p = 7.6 × 10^−4^; Student’s t-test) but not from that of the control (PRE: 0.080 ± 0.05; POST: 0.14 ± 0.03; p = 0.35) condition (Figure 4D). The increase under LSD was consistent in both the LSD_high_ (PRE: 0.10 ± 0.05, n = 9 sessions; POST: 0.44 ± 0.07, n = 8; p = 0.0011) and LSD_low_ (PRE: 0.043 ± 0.05, n = 3; POST: 0.21 ± 0.03, n = 3; p = 0.049) groups. Therefore, consistent with the reduced directionality in individual place cells, LSD reduced the directional specificity of spatial representations at the population level as well.

### Reduced CA1-VC interactions during running

Our results so far indicate that LSD degraded spatial representations in CA1, with reduced firing rates and reduced directionality. To understand whether a miscommunication between HP and VC might underlie the degradation, we analyzed interactions between CA1 and VC cells under LSD. For each pair of CA1-VC cells active during running (active pair), we computed a normalized cross-correlogram relative to those computed from the shuffled spikes of the two cells. We then defined coactivity for the pair as the average correlation value within the time bins around the time lag 0 [−0.1, 0.1] s. Higher coactivity means a higher degree of firing together between the two cells during running. We examined whether coactivity values of CA1-VC active pairs were altered between PRE and POST under different conditions.

**Figure 5.**
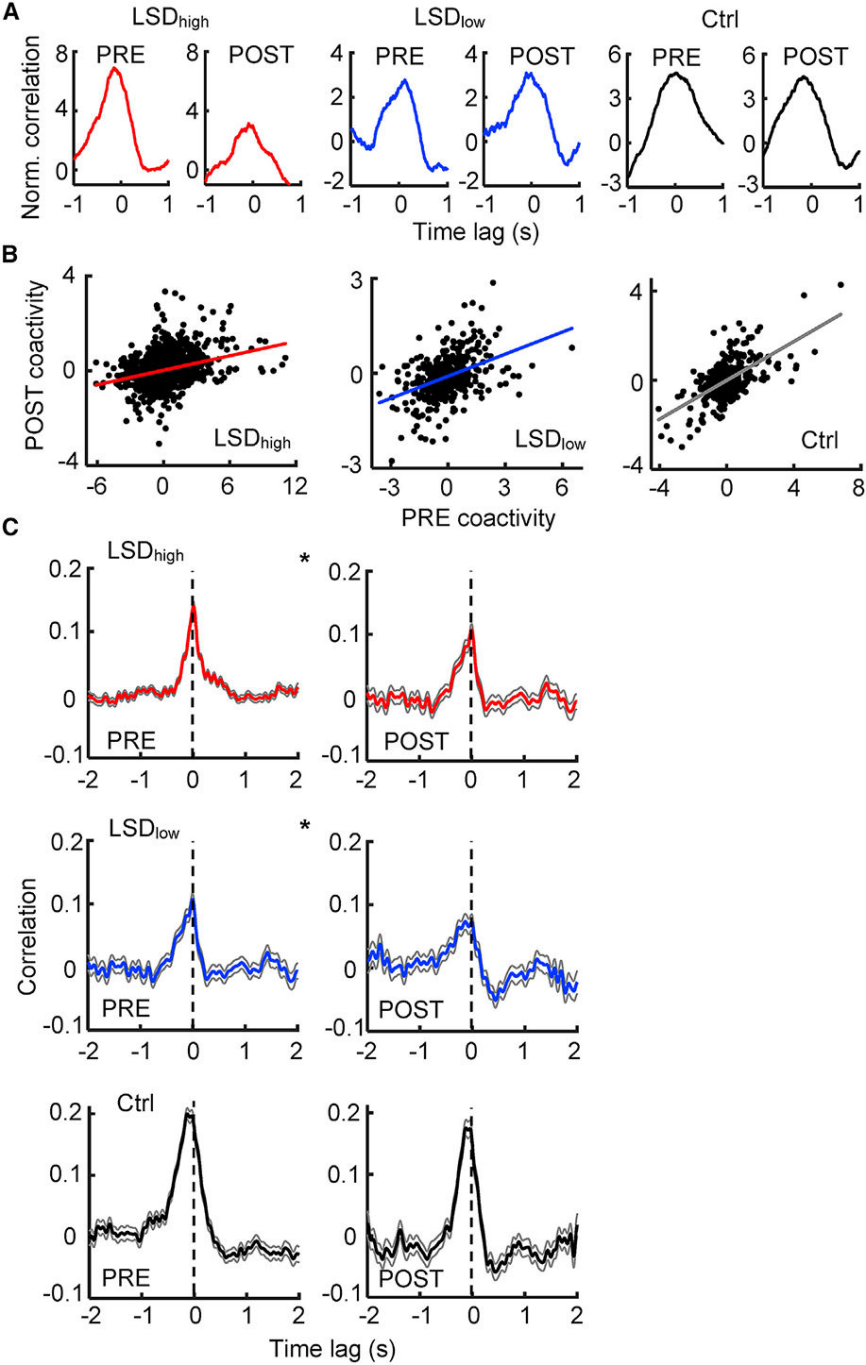
Reduced CA1-VC interaction during running under LSD (A) Example normalized cross-correlograms of CA1-VC active cell pairs during running in PRE and POST under LSD_high_, LSD_low_, and the Ctrl conditions. Note high coactivity values (around time lag 0) in PRE and POST. (B) Coactivity values for all CA1-VC active pairs during running in PRE and in POST under LSD_high_ (n = 1573 pairs), LSD_low_ (n = 396), and the Ctrl condition (n = 305). Each dot is a pair. Line, linear regression between PRE and POST. (C) Average (mean ± SE) cross-correlograms between CA1-VC MUAs over all running periods in PRE and in POST under LSD_high_(PRE: n = 694 running periods, POST: n = 252), LSD_low_ (PRE: n = 252, POST: n = 157), and the Ctrl condition (PRE: n = 230, POST: n = 148). Vertical dashed line, time lag 0. *p < 0.05 for comparing PRE and POST peaks. See also Figure S5.

Examples of normalized cross-correlograms suggest that the cell pairs with high coactivity in PRE maintained high coactivity in POST under all conditions, but with relatively lower peaks especially under LSD_high_ (Figure 5A). Indeed, the distribution of coactivity was narrowed from PRE to POST under LSD_high_ and LSD_low_, but it was unchanged under the control condition (Figure S5A). The narrower distribution could be due to overall changes in firing rates from PRE to POST under LSD.

To examine more specific alterations in coactivity beyond the effects of rate changes, we computed a correlation between coactivity values in PRE and POST for all active pairs. There was a significant correlation in coactivity between PRE and POST under all conditions (LSD_high_: p = 6.3 × 10^−33^, Pearson’s r; LSD_low_: p = 4.7 × 10^−18^; control: p = 3.1 × 10^−33^; Figure 5B), suggesting that the VC-CA1 coactivity patterns overlapped significantly between PRE and POST. However, the correlation was significantly smaller under LSD_high_ (*R* = 0.29, p = 2.1 × 10^−11^, Fisher’s exact test) and under LSD_low_ (*R* = 0.42, p = 1.6 × 10^−4^) than that under the control (*R* = 0.62) condition. This result was true even when the CA1-VC active pairs were downsampled with a matched number of pairs and similar firing rates across the three conditions (Figure S5B). The result indicates that the PRE/POST coactivity patterns were less overlapped under LSD than under the control. Therefore, more changes in CA1-VC interactions had occurred from PRE to POST under LSD.

To further understand how the CA1-VC interaction changed, we examined the multiunit activities (MUAs) in CA1 and VC, which included all sorted and unsorted spikes recorded in an area. We computed the cross-correlation between normalized CA1 and VC MUAs during running. The average cross-correlogram in PRE, over all running periods and all rats under the same condition, had a clearly defined peak under LSD_high_, LSD_low_, and control (Figure 5C). However, the peak was reduced in POST under LSD_high_ and LSD_low_ but not under the control condition (Figure 5C). The mean correlation value at the peak was significantly lower in POST under LSD_high_ (PRE: 0.14 ± 0.01; POST: 0.11 ± 0.01; p = 0.015; Student’s t-test) and LSD_low_ (PRE: 0.11 ± 0.01; POST: 0.073 ± 0.01; p = 0.035) but not under the control (PRE: 0.20 ± 0.01; POST: 0.18 ± 0.01; p = 0.11) condition. The result suggests that LSD reduced the strength of CA1-VC interactions.

### Altered oscillatory activities during immobility

**Figure 6.**
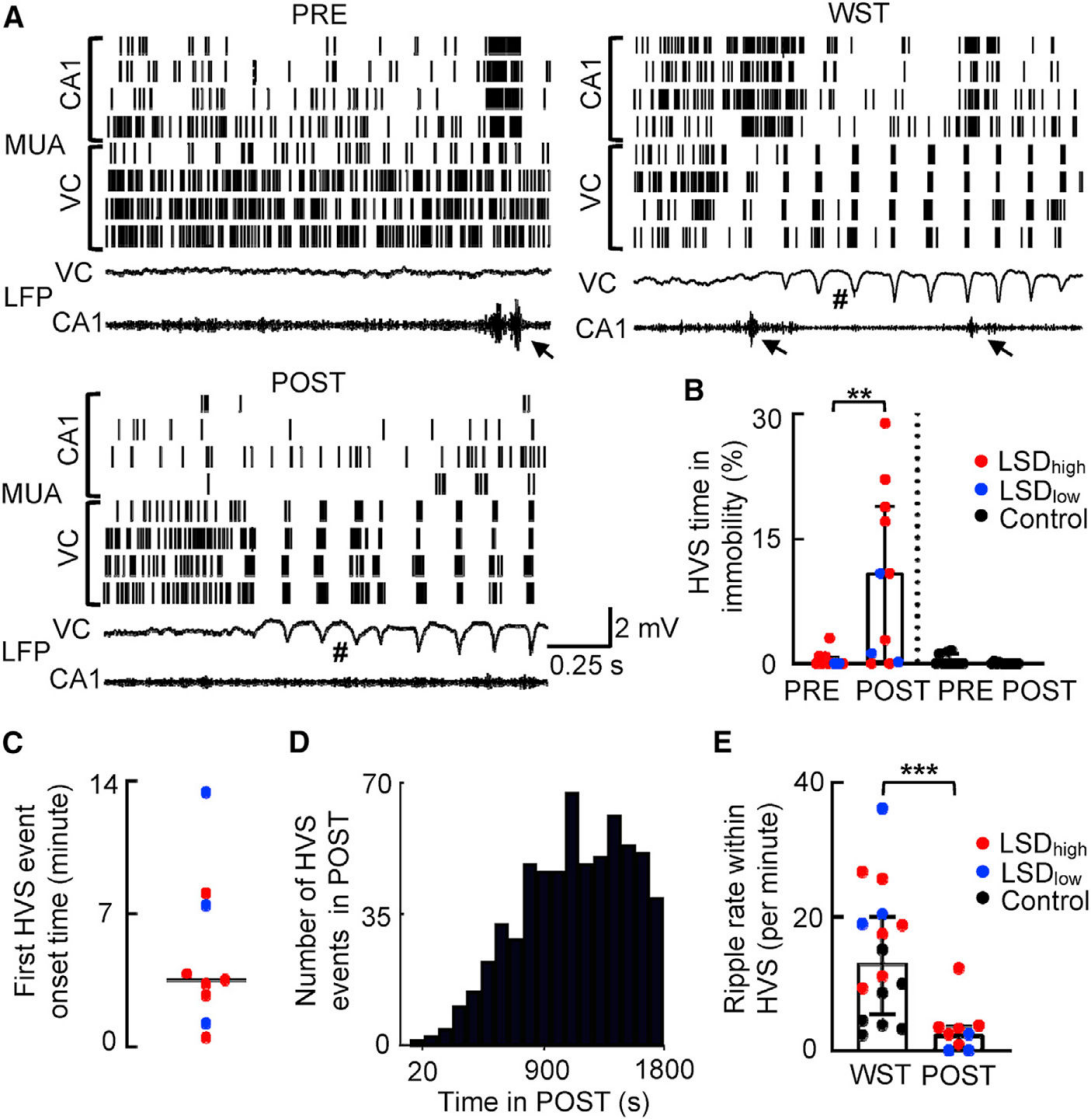
LSD promoted high-voltage spike (HVS) events during immobility (A) CA1 and VC MUAs (top) and LFPs (bottom) in PRE, in WST, and in POST during immobility under LSD_high_ in an example rat. Each row in MUAs includes all spikes recorded from a tetrode. Each tick is a spike. VC LFPs were in broad band (0.5 Hz–2 kHz) and CA1 LFPs in ripple band (100–250 Hz). Arrow, ripple event; #, HVS event. (B) Percentage of time in HVSs among total immobility time in PRE and POST under LSD (n = 11 sessions) and the Ctrl (n = 11) condition. Each dot is a session. Box and bars, median and [25% 75%] range values. (C) Onset times of the first HVS event in POST for each of the 9 rats (out of 11 rats; 2 had no HVSs within the first 30 min of POST). Line, median value. (D) Distribution of HVS peak times in POST under LSD (n = 622 HVS events from 9 rats). (E) Same as (B), but for occurrence rate of ripples per minute within HVS events in WST (n = 16 sessions) and in POST (N = 9) during immobility under LSD. *p < 0.05; **p < 0.01; ***p < 0.001. See also Figure S6.

After analyzing neural activities during running, we switched to the immobile behavior on the track, given the significantly enhanced immobility with LSD (Figure 1F). As expected, the CA1 LFPs displayed ripple events in PRE and POST under all conditions, which were accompanied by bursts of spikes in MUAs of CA1 cells (Figure 6A). The occurrence rate of ripples was reduced from PRE to POST under LSD with small changes in ripple frequency, duration, and amplitude (Figure S6A). Unexpectedly, the VC LFPs frequently displayed a high-amplitude spike-and-wave event (Figure 6A; Figure S6B), called high-voltage spikes (HVSs), which naturally occurs during the wake-fulness-to-sleep transition (WST) in rodents (Haggerty and Ji, 2014; Kandel and Buzsáki, 1997). Here, HVSs were observed during immobility on the track under LSD and accompanied by highly synchronized bursts in MUAs of VC cells (Figure 6A).

We analyzed properties of individual cortical HVS events in PRE and POST, as well as those in WST (in a sleep box prior to the LSD injection). For this analysis, we used cortical LFPs recorded from VC in 12 rats and those recorded from the anterior cingulate cortex (ACC) in 5 rats. Previous studies show that HVS events are highly synchronized across many cortical areas, including sensory and frontal cortices (Sakata et al., 2005; Shaw, 2004). Because our purpose was to examine the occurrence and properties of HVSs under LSD compared to those naturally occurring during WST, we combined the animals with cortical LFPs in VC and ACC and performed within-animal comparisons in HVS properties across sessions. To quantify HVS occurrence, we computed the percent of time in HVSs among total immobility time in PRE and POST. The percentage of time in HVSs was dramatically increased in the first 30 min of POST from that in PRE under LSD (PRE: 0.0% [0.0%, 0.82%]; POST: 11% [0.21%, 19%]; p = 0.0039, Wilcoxon rank-sum test) but not under the control (PRE: 0.0% [0.0%, 1.2%]; POST: 0.0% [0.0%, 0.0%]; p = 0.25) condition (Figure 6B). The first HVS event occurred as early as 48 s on the track with a median onset time of 5.9 [3.3, 13.0] min in the 9 rats (out of 11) under LSD that displayed HVSs within the first 30 min of POST (Figure 6C). The HVS occurrence plateaued around 12 min from the POST start (Figure 6D). Furthermore, we did not observe any transition to slow-wave sleep (SWS) on the track. The rapid HVS onset and lack of SWS indicate that the occurrence of HVSs was not simply due to the rats falling asleep on the track. We compared properties of HVS in POST under LSD to those occurring in WST without LSD. Judging from their waveforms (Figure S6B), the HVSs in POST appeared qualitatively similar to those in WST. Quantifying individual HVS events under LSD in POST did not reveal a significant difference in their median amplitude, duration, or frequency from those in WST (Figure S6C).

Given the prominence of ripples in CA1 and HVSs in the cortex, we asked how the two types of events interacted. We first examined the interaction at the broad LFP and MUA levels. Consistent with our previous study showing weak CA1-VC interactions during WST and unlike SWS (Haggerty and Ji, 2014; Sirota et al., 2003), the cross-correlogram between CA1 LFPs in the ripple band and VC LFPs in the HVS band did not show an obvious peak in WST, and this result was not altered in POST under LSD (Figure S6D). Similarly, the average VC MUAs triggered by CA1 ripple peak times did not show a clear response in WST or in POST under LSD (Figure S6E). We then counted the number of ripples within each HVS event and computed the ripple occurrence rate per minute of HVS. We found a significant reduction in ripple rate within HVSs in POST under LSD from that in WST (WST: 13 [5.5, 20] ripples per min; POST: 2.5 [0.4, 3.7] ripples per min; p = 6.0 × 10^−4^; Mann-Whitney test; Figure 6E). Therefore, LSD during immobility promoted a cortical state similar to WST but with an even weaker interaction with CA1 ripples, consistent with the reduced CA1-VC interaction under LSD during running.

### Maintained pairwise awake reactivation during immobility

It is known that place cell activities during running are replayed within ripples during normal immobility on the track (awake replay), which can be quantified by a pattern analysis like Bayesian decoding that requires a large number of place cells (Davidson et al., 2009; Karlsson and Frank, 2009; Zhang et al., 1998). In our experiments, the number of cells simultaneously active on a trajectory was limited, especially under LSD_high_ when CA1 rates were reduced. Therefore, we used a pairwise approach as in previous studies (Hoffman and McNaughton, 2002; Wilson and McNaughton, 1994). For all pairs of CA1 cells active during running and with non-zero rates within ripples, we computed a correlation between their coactivities during running and those within ripples in the same session. A significant correlation means that cell pairs co-activated during running also co-activated within ripples, which is referred to as awake reactivation.

**Figure 7.**
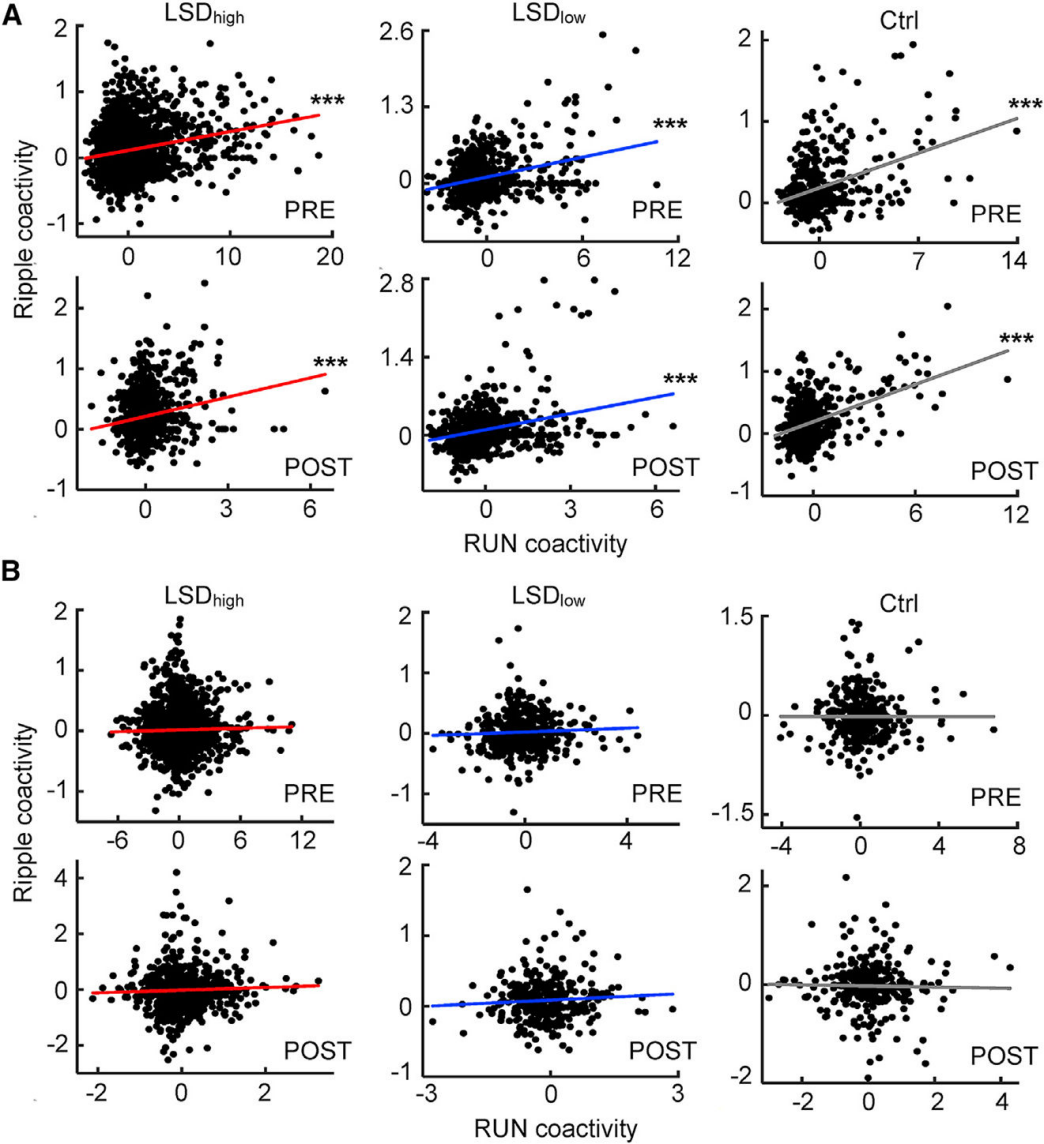
Awake reactivation within CA1 cell pairs but not cross CA1-VC pairs (A) Coactivity during running and within ripples for CA1 cell pairs in PRE and POST under LSD_high_ (PRE: n = 2,479 pairs, POST: n = 921), LSD_low_ (PRE: n = 660, POST: n = 734), and the Ctrl condition (PRE: n = 457, POST: n = 495). Each dot is a pair. Solid line, linear regression. (B) Same as (A), but for cell pairs across CA1 and VC around ripples in LSD_high_ (PRE: n = 1,744, POST: n = 1,075), LSD_low_ (PRE: n = 401, POST: n = 326), and the Ctrl condition (PRE: n = 313, POST: n = 307). ***p < 0.001. See also Figure S7.

We found that awake reactivation of CA1 pairs occurred in PRE and POST under all conditions (Figure 7A). There was a significant correlation between running and ripple coactivities in PRE and POST under LSD_high_ (PRE: *R* = 0.23, p = 2.2 × 10^−30^, Pearson’ r; POST: *R* = 0.20, p = 7.5 × 10^−10^), LSD_low_ (PRE: *R* = 0.32, p = 2.7 × 10^−17^; POST: *R* = 0.29, p = 4.0 × 10^−16^), and the control (PRE: *R* = 0.38, p = 3.0 × 10^−17^; POST: *R* = 0.44, p = 2.8 × 10^−25^) conditions. The correlation values did not significantly differ between PRE and POST under LSD_high_ (p = 0.24, Fisher’s exact test), LSD_low_ (p = 0.29), or the control (p = 0.12) condition. The same result was observed even when the CA1 pairs were downsampled to match the number of pairs and firing rates between PRE and POST (Figure S7A). Thus, awake reactivation during ripples in CA1 persisted under LSD.

We also examined whether such awake reactivation occurred across CA1-VC cell pairs. For this analysis, to account for the possibility that VC cells might fire spikes preceding or lagging CA1 ripples, we computed the cross-correlogram between a CA1-VC cell pair within a time window of [−1, 1] s from each ripple trough time and took the average value with the time lag window [−100, 100] ms as their coactivity. Our analysis found no awake reactivation across the two areas (Figure 7B). There was no significant correlation between running and ripple coactivities for CA1-VC pairs in PRE or POST under LSD_high_ (PRE: *R* = 0.022, p = 0.35, Pearson’ r; POST: *R* = 0.044, p = 0.15), LSD_low_ (PRE: *R* = 0.052, p = 0.30; POST: *R* = 0.069, p = 0.21), or the control (PRE: *R* = −0.0012, p = 0.98; POST: *R* = −0.021, p = 0.71) condition. The same was observed when the CA1-VC cell pairs were downsampled (Figure S7B). Furthermore, such a lack of awake reactivation was also observed within HVS events in POST under LSD_high_ (Figure S7C). Thus, unlike cell pairs within CA1, the cells across CA1 and VC did not coordinately reactivate in ripple or HVS events, suggesting that the awake reactivation within CA1 under LSD was isolated without proper interactions with VC.

## DISCUSSION

To understand how LSD dissociates a subject’s internal perception from the external environment, we have investigated LSD-induced alterations in HP place cell activities and their interactions with VC neurons in freely behaving rats. As rats actively ran on a track, LSD_high_ and LSD_low_ lowered the firing rates of CA1 place cells and reduced their directionality at both the individual cell and ensemble levels. Despite the overall reduction of firing rates in CA1, both CA1 and VC cells temporarily increased their rates before and during HT events. Importantly, the interaction between CA1 and VC neuronal activities was reduced by LSD during running at both doses. During immobility on the track, LSD promoted a cortical state similar to that of WST, which was observed by the occurrence of cortical HVSs. However, different from WST, LSD in immobility further weakened interactions between ripples and HVSs but left the awake reactivation within CA1 preserved. Therefore, LSD reduces normal communication between HP and the sensory cortex, which consequently degrades the HP cognitive map during active running and promotes a state with isolated HP reactivation during immobility. These findings may contribute to the dissociated perceptions produced by LSD.

Our data support that LSD alters HP spatial representation. The firing rates of CA1 place cells, both mean firing rates and within-field rates, were significantly reduced under LSD_high_ and LSD_low_. The reduction persisted even after accounting for rate changes due to speed modulation. For those place cells that remained active under LSD, their directionality was reduced in both high and low doses, i.e., they tended to fire at the same locations despite the different trajectory directions. As such, place cells became less differentiated between the two directions. Despite these changes, the rate curves of place cells were stable, as measured by spatial correlation, indicating that their firing locations were relatively unaltered. The reduced directionality and stability of place cell activities were also observed at the ensemble level, measured by PV correlations. Therefore, our data suggest that LSD induces a less precise, degraded spatial representation of the external environment.

Our finding suggests that this degraded spatial representation may be due to an abnormal interaction between HP and VC. It is well known that visual information is a crucial modality driving the formation of place fields (Muller, 1996; Muller and Kubie, 1987). During track running, it has been shown that firing activities of CA1 place cells and VC neurons are functionally correlated (Haggerty and Ji, 2015). Our data here show that the coactivity between CA1-VC cell pairs changed more in POST than that in PRE under LSD_high_ and LSD_low_, indicating a miscommunication between CA1 and VC cells. More importantly, the cross-correlations between CA1 and VC MUAs were weaker under LSD_high_ and LSD_low_. Therefore, the CA1-VC interaction involved in place cell activities during running is likely reduced by LSD.

The reduced functional interaction between CA1 and VC cells appears to occur during immobility as well. When animals stop on a track, ripple events take place with highly synchronized population bursts of CA1 place cells. We found that ripple rates and other parameters were modestly altered by LSD. In the cortex, we found that LSD dramatically enhanced the occurrence of HVSs, a type of event that normally occurs in WST as the brain shifts to a more internally generated state (Haggerty and Ji, 2014; Kandel and Buzsáki, 1997). During HVSs, cortical neurons are highly synchronized, whereas CA1 ripples occasionally occur. Our previous study suggested that HVSs and ripples during WST produce a weak, transitional interaction between the cortex and HP that eventually leads to a strong correlation between the two areas for memory consolidation in SWS (Haggerty and Ji, 2014). Here, we found that there were fewer ripples during HVS under LSD, suggesting a reduction in cortical-hippocampal consonance. In addition, CA1 cells had reduced firing rates during immobility under LSD_high_ and LSD_low_, and VC neurons also had lower rates with the high dose. Thus, like during running, the CA1-VC communication during immobility is also reduced by LSD. In addition, our data confirm awake reactivation of CA1 activity patterns within ripples during immobility but provide no support for awake reactivation for the joint activity patterns between CA1 and VC. Importantly, LSD_high_ and LSD_low_ did not alter the lack of CA1-VC reactivation in our experiment. Because LSD enhanced immobility on the track, this finding further supports the idea that LSD reduces CA1-VC communication, namely, this time through the promotion of a behavioral state lacking coordinated reactivation, which possibly leads to isolated CA1 awake reactivation in the absence of engagement from the sensory cortices.

Our study helps to understand how neural activity changes during the HT behavior. HT is believed to be a behavioral signature of a brain state similar to hallucination in humans, because the 5HT_2A_R agonists that are hallucinogenic in humans also evoke HT in rodents, whereas those 5HT_2A_R agonists that are non-hallucinogenic in humans do not evoke HT (Fantegrossi et al., 2008; González-Maeso et al., 2007). Our analysis indicates a temporary increase in the firing rates of CA1 and VC cells when HT occurred, despite the overall reduction in the firing rates in POST under LSD. This increase was broad, starting seconds earlier than and lasting beyond HT events. We did not find specific, short-term changes immediately before HT in our data. However, it is possible that there exist signals in other brain areas that are more tightly correlated with HT. Nevertheless, based on our data, we speculate that the increase in firing rates is a temporary compensation for the overall rate reduction under LSD and that HT is a coping behavior in response to internal percepts associated with the temporarily increased neural activities in HP and VC.

One of our findings is that LSD greatly promotes the occurrence of HVSs. Because HVSs naturally occur during WST (Haggerty and Ji, 2014; Kandel and Buzsáki, 1997), we propose that LSD produces a WST-like state on the track as animals become less engaged with the task and descend into immobility. Our analysis shows similar quantitative properties between LSD-induced and WST HVSs, suggesting that the LSD-induced state is similar to WST. However, unlike the HVS events in WST that are normally followed by SWS, we did not detect any SWS on the track even when strong HVSs occurred repeatedly under LSD. The observation suggests the cortex perpetuates a highly synchronized state without going to sleep. It is unknown how this cortical state is related to LSD-induced hallucinations. However, hypnagogic imagery does occur often in WST in humans (Stickgold et al., 2000), and a recent study shows that slow oscillatory events in the retrosplenial cortex are involved in the effect of dissociative drugs such as ketamine (Vesuna et al., 2020). In addition, the forward propagation of alpha waves, a potential signature of HVSs in humans, is enhanced by the hallucinogen N- Dimethyltryptamine or DMT (Alamia et al., 2020). It is possible that this LSD-induced state similar to WST may contribute to hallucinations under LSD.

A previous human fMRI study shows that activities in the parahippocampal gyrus are reduced and the functional connectivity in the hippocampal-prefrontal network is disrupted by LSD in an active task (Schmidt et al., 2018). Another study found that the functional connectivity between HP and VC is lower in patients with Parkinson’s disease who display visual hallucinations (Yao et al., 2016). These findings are consistent with our results that CA1 neurons had lower firing rates and lower functional interaction with VC during running. When humans rested awake with eyes closed, fMRI studies found that VC is hyperactive under LSD, with enhanced functional connectivity with some brain areas (Carhart-Harris et al., 2016; Müller et al., 2018), which seems inconsistent with our result that VC neurons reduced their rates during immobility. However, our finding may not necessarily contradict human studies. It is unclear whether the resting state in humans with eyes closed is the same as immobility in rodents. Second, it is also unclear whether the increase in fMRI signals directly translates to an increase in firing rates of individual neurons. It is possible that large LFP signals such as HVS events under LSD underlie the increased fMRI signals (Logothetis et al., 2001; Magri et al., 2012). Finally, the dosage of LSD used in rodent studies including ours (60 μg/kg or 240 μg/kg) is much higher than that in human studies (single dose of ~100 μg), which could lead to differences in neurophysiological responses.

Overall, our work reveals neurophysiological alterations that can advance our understanding of how LSD produces its powerful reality-altering effects. The reduced HP-VC interaction leads to degraded spatial representations during active tasks, resulting in an altered cognitive map different from the external environment. The temporary, compensatory increase in firing rates of VC and HP cells around HTs may further alter the sensory and memory processing under LSD. Furthermore, LSD prolongs the immobility behavior and promotes a WST-like state with enhanced cortical HVSs, during which HP reactivates spatial representations in isolation without the participation of the sensory cortex. Our findings contribute to the neural circuit mechanism of LSD-induced hallucinations by identifying a specific functional dissociation between sensory and memory circuits through VC and HP miscommunication, which may produce abnormal spatial representations of the external world and/or abnormal sensory percepts misaligned with external reality.

## STAR★METHODS

### RESOURCE AVAILABILITY

#### Lead contact

Further information and requests for resources should be directed to and will be fulfilled by the Lead Contact, Daoyun Ji (dji@bcm.edu).

#### Materials availability

This study did not generate new unique reagents.

#### Data and code availability

- The analyses in this paper were performed by MATLAB scripts with existing MATLAB functions. The MATLAB codes for the main analysis tool are publicly available in GitHub (https://gitbub.com/DaoyunJiLab/DataManager).
- Any additional information required to reanalyze the data reported in this paper is available from the lead contact upon request.
- Data reported in this paper will be shared by the lead contact upon request.

### EXPERIMENTAL MODEL AND SUBJECT DETAILS

Twenty-three male Long-Evans rats between 4 and 9 months of age were used in this study. Each rat was individually housed, food-restricted with weight maintained at or above 85% of *ad libitum* level, and trained to run on a track for food rewards (pre-training). Electrophysiological recordings of neuronal activities and/or behavioral experiments were conducted in the 23 rats under various drug conditions (see below). All animal and research procedures adhered to the “Guide for the Care and Use of Laboratory Animals” of the National Institute of Health and were approved by the Baylor College of Medicine Institutional Animal Care and Use Committee.

### METHOD DETAILS

#### Surgery

For the electrophysiological recording experiment, each rat was surgically implanted with a custom-built microelectrode array (tetrode drive) under anesthesia with 0.5 – 3% of the inhalation isoflurane. The tetrode drive included 24 - 32 movable tetrodes that intended to target one or two recording areas. The CA1 of dorsal hippocampus at coordinates anterior-posterior (AP) −3.8 mm and medio-lateral (ML) 2.4 mm to the Bregma was a target in all 23 rats (not everyone yielded useful electrophysiological data; see Table S1). The other targeted area was either the visual cortex (VC, 15 rats) at AP 6.8 mm and ML 4.5 mm or the anterior cingulate cortex (ACC, 5 rats) at AP 1 mm anterior and ML 1 mm. For the ACC recording site, only the LFPs were used for analyzing cortical high-voltage spikes (HVS); single-unit ACC data were not used in this study. A bipolar electrode made from a steel wire (0.11 mm diameter; A-M Systems) was inserted into the neck muscle to record electromyography (EMG). The tetrode drive was anchored to the skull using stainless steel screws and dental cement. The analgesic buprenorphine (slow-release) or ketophen was administered subcutaneously prior to surgery to assist with recovery. Animals did not resume training or food restriction until fully recovered from the surgery and tetrodes reached the targets, about 2 - 4 weeks post-surgery.

#### Behavioral procedure and drug conditions

Animals were pre-trained to run back and forth (two trajectories) for food reward (condensed milk) along a C-shaped track, which was 3.5 m long. The pre-training lasted for at least 4 days until they reliably and consistently ran at least 20 laps on the track. After the pre-training, a behavioral procedure was conducted for one or more days per animal. Each daily procedure began with a rat resting in a sleep box followed by a subcutaneous injection of 0.5 mL saline. Fifteen minutes following the injection, the rat ran for food rewards back and forth along two trajectories on the now familiar (after pre-training) C-shaped track for 20 - 30 minutes (PRE). The rat then slept in the sleep box for ~3 hours between sessions, followed by another administration of 0.5 mL under various drug conditions. Fifteen minutes after the second drug administration, the animal performed the same task for 20 – 70 minutes on the track (POST). The variation in the duration of POST was needed to collect sufficient number of running laps (≥6) in a session, especially for the animals injected with LSD_high_ who’s running activities were greatly reduced. However, longer sessions might lead to increased immobility behavior or immobility-related neural activities that were drug-irrelevant. To eliminate this possibility, we restricted our analysis on immobility-related neural activities within the first 30 minutes in POST.

The drug condition for the second administration was the injection of a high dose of LSD at 0.24 mg/kg (LSD_high_), a low dose of LSD at 0.06 mg/kg (LSD_low_), or one of the control injections including the injection of saline alone, the injection of a 5HT_2A_R antagonist M100907 alone at 0.2 mg/kg, or the injection of M100907 at 0.2 mg/kg together with the low dose of LSD at 0.06 mg/kg. In the M100907 and LSD together condition, M100907 was administered 15 minutes prior to the injection of LSD. M100907 at this concentration is known to block 5HT2ARs at even high concentrations of LSD (Nichols, 2004). If an animal was used in this procedure for more than one day, the condition on the first day was always saline or M100907 (Table S1). Electrophysiological recordings were performed during this behavioral procedure in 23 rats across 28 days of experiments. The other 2 days’ experiments did not have neural recordings, but their behavioral data were included in the behavioral analysis.

#### Electrophysiological recording

Recordings were performed using tetrodes made by twisting 4 fine nichrome wires (diameter 13 μm; Sandvik Palm Coast, Palm Coast, FL), as previously described (Haggerty and Ji, 2014, 2015). During the two to four weeks post-surgery, tetrodes were descended slowly to the target regions: CA1, VC, or ACC. Neuronal spikes and LFPs, as well as the EMGs, were acquired using a Digital Lynx system (Neuralynx, MT). For the ACC recordings, only the LFPs were used in this study for analyzing cortical HVS events. In 19 animals, LFP signals were sampled at 2 kHz with a broad-band filter (0.1 – 1 kHz); in other 4 animals LFPs were sampled at 4 - 8 kHz. Spikes were identified using a preset threshold of 50 - 70 μV from signals filtered within 600 Hz - 9 kHz and sampled at 32 kHz. Animal positions were tracked using two diodes mounted to the tetrode drive and recorded by an overhead camera. Position data were sampled at 33 Hz with a resolution of ~0.25 cm per pixel.

#### Histology

After the recording, animals were euthanized by pentobarbital overdose (200 mg/kg). For recording site identification, electrical lesions were made at each recording site by passing a current of 30 μA for 15 s. Brains were fixed with 10% formalin and sectioned at 90 - 300 μm thickness. Sections were stained with 0.2% cresyl violet or 1% sodium sulfide nonahydrate (for acetylcholineesterase activity). Recording locations in CA1 and ACC were verified from the lesion marks in the cresyl violet stained sections. Recording sites in VC were determined from the sodium sulfide nonahydrate staining as shown in previous studies (Haggerty and Ji, 2014, 2015).

### QUANTIFICATION AND STATISTICAL ANALYSIS

For the majority of analyses on neuronal activities, we considered LSD_high_ and LSD_low_ conditions separately because the number of neurons recorded under each condition (LSD_high_: N = 435 CA1 cells, N = 99 VC cells; LSD_low_: N = 177 CA1 cells, N = 22 VC cells; Table S1) was sufficient for the study of dose-dependent responses to LSD (see details below). However, for behavioral analyses and certain neuronal analyses that required individual rats as samples, we combined LSD_high_ and LSD_low_ together, due to the limited number of animals in each condition (LSD_high_: N = 10 rats, LSD_low_: N = 4 rats). Although the combination did not permit the study of dose-dependent responses in these measures, it still allowed us to examine the effects of LSD in comparison to the control. For various drug conditions in the control group, behavioral and neuronal activity results were similar and thus combined unless specified otherwise.

Statistical details of the experiments including *N*, statistical tests, and their descriptive statistics are found in the Results section of the paper. Significance is defined in the figure legends as *p < 0.05; **p < 0.01; ***p < 0.001.

#### Behavioral quantifications

The behavior of an animal during tracking running was quantified for each session by the number of running laps, running speed, the amount of time the animal was immobile, and the number of head twitches (HTs).

A running lap was the time period when the animal ran from one end of the track to the other. Instantaneous running speed was calculated for every time point using the position data and smoothed by a Gaussian window with a sigma of 0.5 s. Slow-speed or stopping periods were removed from speed calculations by removing events when the animal’s speed was below 10 cm/s for at least 0.5 s. Immobility periods throughout the entire session including the ends of the trajectory were used for ripple analysis and percent of immobility in a session. These periods were identified by setting a speed threshold below the mean speed at the reward sites, with the same threshold applied to PRE and POST for each animal. If two neighboring immobility periods (either within or outside the running laps) had a gap smaller than 0.5 s, they were combined into a single immobility period.

A head twitch (HT) was visually identified from the recorded videos of the running sessions. Across most animals, two experimenters independently scored and compared counts for reliability. In addition to visual observation, in a subset of sessions (N = 8) under LSD_high_ or LSD_low_, the start and end times of each HT was precisely determined automatically from deflections in the filtered EMG signal recorded from the rat neck muscle. These EMG-identified HTs were referred to as eHTs. In this case, raw EMG signals were band-pass filtered within 20 - 60 Hz to remove the movement-related artifact. eHTs were detected when at least 2 peaks exceeded 8 standard deviations (SDs) from the filtered trace baseline with a minimum inter-peak time of 0.1 s. The eHT start and end times were assigned when the signal first and last exceeded a threshold of 3.5 SDs.

#### Single-unit dataset

Our analyses on single-unit data were performed on a total of 781 CA1 neurons and 153 VC cells from 17 rats on 20 recording days (Table S1). Single units were sorted offline using custom software (xclust, M. Wilson at MIT, available at GitHub repository: https://github.com/wilsonlab/mwsoft64/tree/master/src/xclust). The VC cells were mostly located in the primary visual cortex V1 (140) and the rest (13) were located in the neighboring secondary visual cortex. We identified those CA1 and VC cells that were active on at least one trajectory in at least one of the track-running sessions (PRE or POST) with a minimum firing rate of 0.5 Hz (active cells). CA1 silent cells were defined as those with rate < 0.5 Hz and putative interneurons in the CA1 as firing rate > 5 Hz in both trajectories during running. Firing rates of CA1 and VC active cells, CA1 silent cells, and CA1 putative interneurons were analyzed during running and during immobility. For VC cells, we did not analyze those silent (< 0.5 Hz; N = 15) or those with very high rate (> 25 Hz, N = 8) due to their small numbers. Further analyses described below were only performed on active CA1 (N = 365) and active VC (N = 130) cells.

#### Quantification of spatial firing properties

We analyzed the spatial firing properties of active CA1 cells. Spikes occurring at any stopping periods were excluded from the analysis. For each cell on its active trajectory in a session, we constructed a firing rate curve by computing the average firing rate across all laps at each position along the trajectory, excluding the positions within ~10 cm from the reward sites. The rate curves had a spatial bin size of 1 cm and were smoothed using a Gaussian window with a sigma of 3 bins. Spatial information (SI) was computed from the firing rate curve according to the established formula as in previous studies (Haggerty and Ji, 2015; Skaggs et al., 1993). Stability was the Pearson correlation between the two firing rate curves on the same trajectory between PRE and POST. For this calculation, a cell was included if it was active on the trajectory in at least one session (rate > 0.5 Hz) and fired at least one spike in the other session. Directionality was measured by a Pearson’s correlation between the two firing rate curves on the two trajectories in the same session. In this case, a cell was included if it was active on one trajectory (rate > 0.5 Hz) and fired at least one spike on the other.

For a CA1 cell on an active trajectory in a session, its place fields were identified using a threshold of 3 Hz for peak rates. Boundaries of a place field were determined by 10% of its peak rate. Fields with a gap smaller than 5 cm were combined. The within-field firing rate of a field was the mean rate of the rate curve within the field’s boundaries.

For comparing firing rates between PRE and POST sessions, we removed the effect of running speed on firing rates. In this case, for each cell active on a trajectory we computed its mean firing rate *r_i_* and mean running speed *s_i_* during every lap *i* on the trajectory in both sessions. We performed a linear regression between the lap-by-lap rate and speed: *r* = *ks* + *b*. We then computed a residual rate *r_i_*‘ = *r_i_* − (*ks_i_* + *b*). The residual firing rate for PRE or POST was the average value of *r_i_*‘ for those laps in PRE or POST. The within-field rates were similarly corrected for the effect of speed. In this case, the lap-by-lap firing rate and speed were computed within the boundaries of each place field. In addition, we computed a burst index for CA1 active cells, which was the percentage of spikes of a cell during running that occurred within 10 ms of another spike.

#### Theta oscillation, theta phase precession and theta sequence

To compute theta peak frequency and theta power, we divided running laps of a session into different events with different running speeds (< 20, 20 −40, 40-60, > 60 cm/s) with minimum length of 2 s. We then used multi-taper method to estimate the power spectral density (PSD) of CA1 LFPs. Theta peak frequency (at 0.25 Hz resolution) and total theta power were obtained for each speed range of a session. Not all speed ranges were available in a given session, especially for POST under LSD when rats tended to run slower. Coherence was computed between CA1 and VC LFPs during running and then averaged over the theta band (6 – 10 Hz). To obtain theta phases of spikes of a CA1 place cell, CA1 LFPs were filtered within [6 10] Hz and theta peaks and troughs during active running were identified. Theta phases were assigned according to spike times relative to their nearest theta peaks (360°/0°) and troughs (180°). Theta phase properties for cells active in a session were computed according to circular statistics. Theta phase precession for spikes within a place field was quantified by the maximum linear correlation between phases and positions within the field (O’Keefe and Recce, 1993). Only the fields with a minimum of 20 spikes were included in the analysis. To quantify theta sequences (Dragoi and Buzsáki, 2006; Robbe and Buzsáki, 2009), we selected those place cells with the peak distance of their overlapping place fields < 40 cm and identified their cross-correlation peak time as a measure of firing interval within theta cycles. The correlation between the field distance and the theta firing interval among all overlapping place fields in a session was computed and compared between PRE and POST.

#### Population vector (PV) analysis

Population vectors (PVs) were constructed for every spatial bin of a trajectory from rate curves of all cells on a day active on the trajectory. Only PVs at those locations with at least 5 active cells were included in the PV correlation analysis. A PV correlation was the Pearson’s correlation between the two PVs at the same spatial bin either for two sessions (PRE and POST, cross-session PV_corr_) or for two trajectories (cross-trajectory PV_corr_). The PV correlations for all the spatial bins and all animals were combined and compared among different experimental conditions.

#### Pairwise correlation analysis

To quantify pairwise interactions within CA1 cell pairs or between CA1 and VC cell pairs, we computed a normalized cross-correlation of two spike trains, relative to the shuffled spike trains. We first computed a spike-count cross-correlation using the two original spike trains within all events under consideration (e.g., all running laps in PRE, or ripple events in POST). Then, each of the two spike trains was independently shifted by a random time within each event and a cross-correlation was computed using the shuffled spike trains. The shuffling was repeated for 200 times. The mean and standard deviation (SD) were computed at each time bin from all the shuffle-generated cross-correlations. The normalized correlation value at each time lag was the Z-score of the original correlation value relative to the shuffle mean and SD.

For within-CA1 cell pairs, we computed their normalized cross-correlations during running laps, with stopping periods within laps removed. The bin size was 10 ms and the cross-correlation was smoothed by nearest-neighbor averaging among ± 10 bins (100 ms). Coactivity between two cells was the average Z scores around time lag 0 ([−100 100] ms). For within-CA1 cell pairs, we also computed their normalized cross-correlations within ripple events. In this case, the bin size was the same 10 ms, the cross-correlation was smoothed using ± 4 bins (40 ms), and coactivity was the average Z-score among the time lag [−50 50] ms. For CA1-VC pairs, we computed their normalized cross-correlations and coactivities during running similarly as within-CA1 pairs. But for ripple events, the CA1-VC cross-correlation was performed in time intervals of [−1 1] s from the ripple peak times and coactivity was the average Z-score among the time lag [−100 100] ms, to account for the possibility that VC neurons might activate earlier or later than the CA1 ripples. Awake reactivation was identified by correlating coactivities of all cell pairs during running with those within ripples. In this case, we included those cell pairs that contained at least one cell active during running (rate > 0.5 Hz) and the other fired at least one spike during running.

#### MUA analysis

All spikes in a recording area (CA1 or VC) were included in the multiunit activities. The spikes in a given area were counted in 10 ms time bins. The spike counts were normalized to values within [0 1] with 0 meaning no spikes and 1 meaning maximum spike count. The spike counts were then smoothed by a Gaussian kernel with a sigma of 3 bins into a MUA activity curve with time. A cross-correlation between CA1 and VC MUAs was computed within lags of [−1 1] s for running periods longer than 2 s without immobility. The mean MUA cross-correlation for a session under a condition was the average cross-correlation among all running periods in all animals under the same condition. For analyzing fine temporal relationship between VC MUAs and CA1 ripples, we used ripple peak times (time 0) to trigger VC MUAs within ripple events and the average VC MUAs were computed at time lags within [−50 50] ms (to identify possible peaks within an HVS cycle of ~100 ms), relative to baseline periods of [−120 – 100] ms and [100 120] ms.

#### HT-triggered averages

We computed the average velocity, linearized 1-dimensional (1D) position, and firing rates of CA1 and VC cells triggered by the EMG-identified head-twitching (eHT) start times. We aligned the start times of all eHTs in POST under LSD_high_ and LSD_low_ as time 0 s. For triggered averages of velocity and 1D-position, we considered every 50 ms bin (time lag) within a time interval of [−1 1] s around the eHT start times. The HT-triggered average velocity at a time lag was the average across all values of velocity at the time for all eHTs. For HT-triggered average 1D-positions, we aligned the 1D positions at time lag 0 s as 0 cm and averaged all 1D-positions relative to this 0 position at every time lag for all eHTs. For HT-triggered average firing rates, we considered a time interval of [−5 5] s with a bin size of 50 ms. At every time lag, firing rates of all running-active cells in CA1 or VC at the time lag were averaged across all cells and all eHTs.

#### High voltage spike (HVS) and ripple event detection

HVS events were detected as described in previous work (Haggerty and Ji, 2014). Cortical LFP was filtered with a band-pass of 6 - 12 Hz. HVS were identified if the filtered LFP trace exceeded a trough threshold set as 6 SD below the baseline trace with at least 4 troughs below this threshold and with a maximum inter-trough interval of 250 ms. The start and end time was assigned at the point when the signal first and last exceeded a threshold of 2.5 SD. The peak time was determined as the time with the lowest trough amplitude within the HVS event. Neighboring HVS events with a gap between them less than 0.5 s were combined into a single event. Detected events were visually examined to ensure reliable and consistent identification, and parameters may have been slightly adjusted in rare cases in which HVS events were mis-identified.

We detected ripple events from a band-pass filtered (100 - 250 Hz) hippocampal LFPs. Ripple events were identified by a trough threshold exceeding 6 SD from baseline. The start and end times of ripples were identified as the moment the amplitude of the filtered LFP trace crossed 2.5 SD and only ripple events between 30 and 400 ms in duration were included in the analysis. If neighboring ripple events were separated by fewer than 30 ms, they were combined into a single event. In addition, we also computed the cross-correlation between ripple-filtered (100- 250 Hz) CA1 LFPs and HVS-filtered (6 – 12 Hz) VC LFPs as a measure of possible fine temporal relationship between CA1 and VC activities during HVS events.

## Supplementary Material

1

## Figures and Tables

**Table:** KEY RESOURCES TABLE

REAGENT or RESOURCE	SOURCE	IDENTIFIER
Experimental models: Organisms/strains		
Rat: Long-Evans	Charles River Laboratories	Substrain: Crl: RRID: RGD_2308852
Software and algorithms		
DataManager	Daoyun Ji Lab at BCM	https://github.com/DaoyunJiLab/DataManager
Digital Lynx	Neuralynx	https://neuralynx.com/hardware/digital-lynx-sx
xclust	Matthew Wilson Lab at MIT	https://github.com/wilsonlab/mwsoft64/tree/master/src/xclust

## Highlights

- LSD reduces firing rate and directionality of hippocampal place cells
- LSD reduces hippocampal-cortical interactions in active/immobile behavior
- LSD promotes a cortical state similar to wakefulness-to-sleep transition
- Reactivation within hippocampus but not across hippocampus and cortex under LSD
