# Supplementary material for: LSD degrades hippocampal spatial representations and suppresses hippocampal-visual cortical interactions
Source: Cell Rep. Author manuscript; Available in PMC 2022 Dec 29. (PMC9798728; doi:10.1016/j.celrep.2021.109714)
Supplement: 1 [file NIHMS1856541-supplement-1.pdf]

Cell Reports, Volume *36*

Supplemental information

LSD degrades hippocampal spatial representations and suppresses hippocampal-visual cortical interactions

Carli Domenico, Daniel Haggerty, Xiang Mou, and Daoyun Ji

Table S1. **Animals used for experiments under various behavioral conditions, related to STAR Methods**. Data from each animal used in behavioral, LFP and single-cell analyses are listed. CA1: hippocampal CA1 area; V1/V2: primary/secondary visual cortex; ACC: anterior cingulate cortex. HT: head-twitching events detected from video or electromyography (EMG); WST: wakefulness-to-sleep transition periods analyzed from recorded sleep session. -: not available. In several animals, position data were not available, meaning track running behavior was not examined.

Animal name	Behavioral condition	Number of CA1 cells	Number of active CA1 cells	Number of VC cells	Number of active VC cells	LFP	HT	WST
BTT	LSDhigh	39	26	11	9	CA1, V1	Video, EMG	R
Beg	LSDhigh	48	22	9	8	CA1, V1	video	R
CSM	LSDhigh	10	8	6	4	CA1, V1	-	-
Ca	LSDhigh	13	5	13 (V2)	8	CA1, V2	Video, EMG	R
FP	LSDhigh	72	43	16	13	CA1, V1	Video, EMG	R
JZ	LSDhigh	108	44	14	11	CA1, V1	video	-
MB	LSDhigh	70	25	15	13	CA1, V1	-	R
MU	LSDhigh	19	11	6	6	CA1	video	-
RIP	LSDhigh	23	11	6	5	CA1, V1	Video, EMG	R
WKA	LSDhigh	33	12	3	3	CA1	video	-
Or	LSDlow	79	37	10	8	CA1, V1	Video, EMG	R
Tan	LSDlow	35	22	6	6	CA1, V1	Video, EMG	R
WAJ	Saline	56	25	8	8	CA1, V1	Video	R
	LSDlow	63	25	6	6	CA1, V1	Video, EMG	R
Ni	M100907 + LSDlow	7	1	6	5	-	video	-
PT	M100907	16	8	8	7	CA1, V1	video	R
	M100907 + LSDlow	17	10	10	10	CA1, V1	video	R
Pe	M100907 + LSDlow	4	2	-	-	-	-	-
OSI	Saline	20	8	-	-	ACC	video	-
	M100907 + LSDlow	49	20	-	-	ACC	video	-
Total cells		781	365	153	130			
Ringo	Saline	-	-	-	-	ACC	video	-
	M100907 + LSDlow (no position data)	-	-	-	-	ACC	video	-
Beowulf	Saline	-	-	-	-	CA1, ACC	Video	R
	M100907 (no position data)	-	-	-	-	CA1, ACC	video	R
	LSDlow (no position data)	-	-	-	-	-	Video, EMG	-
Kyoto	Saline	-	-	-	-	-	video	-
	M100907 + LSDlow	-	-	-	-	-	video	-
Sully	M100907	-	-	-	-	CA1, ACC	video	R
Jackie	M100907 + LSDlow	-	-	-	-	CA1	video	-
Ozark	Saline	-	-	-	-	CA1, ACC	video	R

Supplemental Figure 1

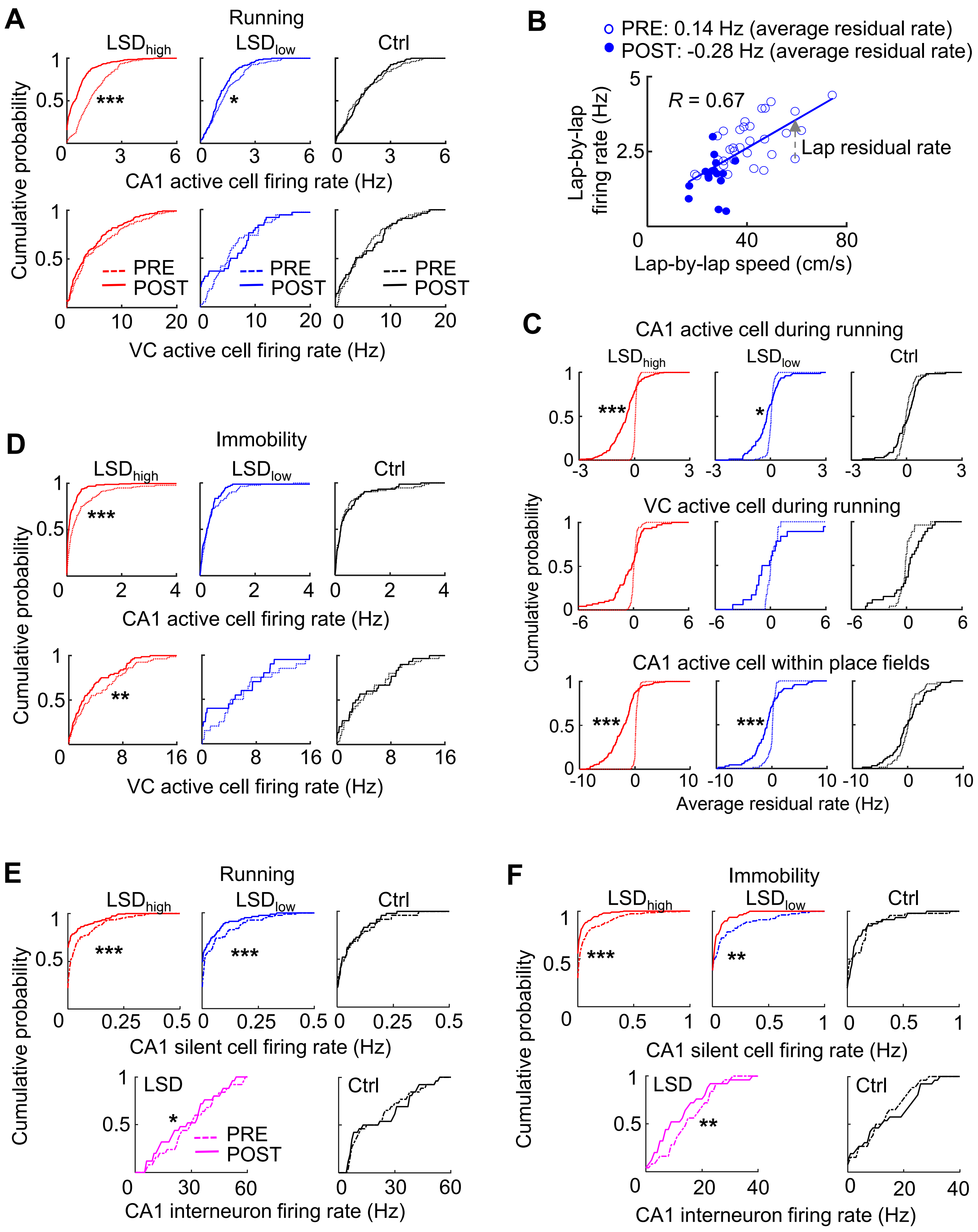

Figure S1. **Firing rate reduction in CA1 and VC cells under LSD during running and during immobility, related to Figures 2&3.**

(**A**) Cumulative distributions of firing rate under LSD_{high}, LSD_{low}, and the control (Ctrl) conditions for CA1 (*N* = 279, 124, 109 cell x trajectories for LSD_{high}, LSD_{low} and Ctrl respectively, same below) and VC (*N* = 150, 38, 57) active cells during running in PRE and POST.

(**B**) Linear regression between lap-by-lap speeds and firing rates for an example CA1 place cell (under LSD_{low}) for all the PRE and POST laps. *R*: correlation value. Residual rate is the difference between actual rate and the regression line, as shown by an example lap (arrow). The average residual rate among all laps in a session, which measures the relative mean rate after removing the speed modulation, is shown for the cell in PRE and POST.

(**C**) Cumulative distributions of average residual rate under LSD_{high}, LSD_{low}, and the control condition in PRE and POS for CA1 (*N* = 198, 79, 69 cells x trajectories) and VC (*N* = 70, 18, 27) active cells during running, and for CA1 active cells within their place fields (*N* = 161, 69, 61 fields).

(**D**) Cumulative distributions of firing rate under LSD_{high}, LSD_{low}, and the control condition in PRE and POST for CA1 (*N* = 207, 84, 74 cells) and VC (*N* = 80, 20, 27) active cells during immobility.

(**E**, **F**) Same as (**A**, **D**), but for CA1 silent cells (*N* = 196, 80, 48 cells) and CA1 putative interneurons (*N* = 18, 7, 22). The interneurons under LSD_{high} and LSD_{low} are combined (LSD, pink), due to their small numbers under these conditions.

The median rates (all in Hz) and *P*-values for silent cells in **E** are the following. LSDhigh: PRE 0.013 [0.0021 0.057], POST 0.0 [0.0 0.013], $P = 1.6 \times 10^{-16}$; LSDlow: PRE 0.012 [0.0017 0.097], POST 0.0024 [0.0 0.050], $P = 4.2 \times 10^{-4}$; Ctrl: PRE 0.034 [0.0023 0.11], POST 0.023 [0.0071 0.10], *P* = 0.76.

The median rates and *P*-values for interneurons in **E** are the following. LSD: PRE 30 [21 43], POST 29 [13 39], *P* = 0.043; Ctrl: PRE 22 [7.8 34], POST 23 [7.4 39], *P* = 0.17.

The median rates and *P*-values for silent cells in **F** are the following. LSDhigh: PRE 0.023 [0.0073 0.094], POST 0.0063 [0.0 0.032], $P = 1.3 \times 10^{-20}$; LSDlow: PRE 0.033 [0.0041 0.13], POST 0.017 [0.0044 0.057], *P* = 0.0066; Ctrl: PRE 0.070 [0.011 0.18], POST 0.060 [0.018 0.15], *P* = 0.56.

The median rates and *P*-values for interneurons in **F** are the following. LSD: PRE 15 [9.9 23], POST 10 [5.5 19], *P* = 0.0041; Ctrl: PRE 12 [4.4 20], POST 13 [4.9 24], *P* = 0.85.

**P* < 0.05, ***P* < 0.01, ****P* < 0.001, comparing median values between PRE and POST by *Wilcoxon signed rank* test.

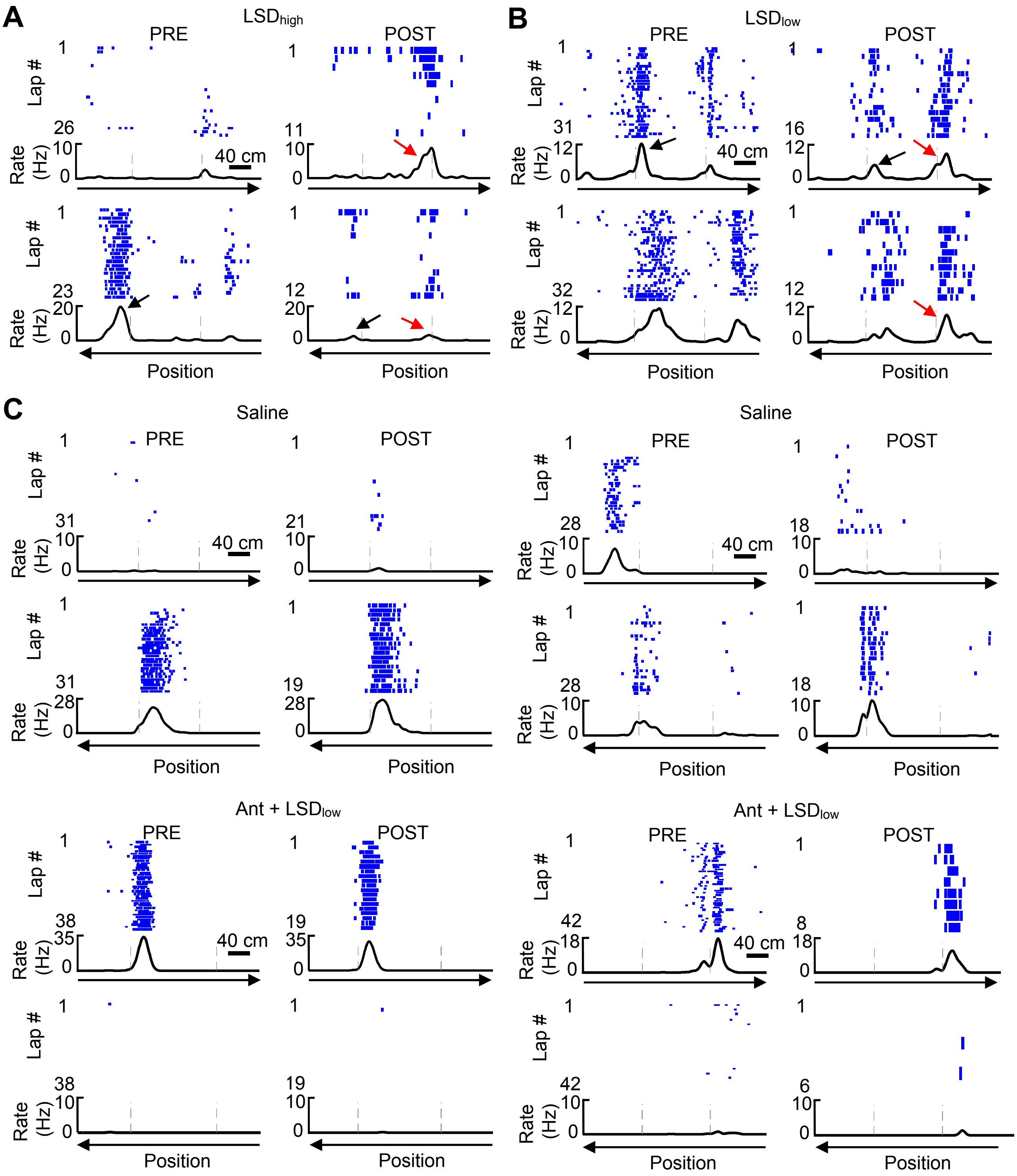

Figure S2. More example place cells under LSD$_{high}$, LSD$_{low}$ and the control condition, each from a different rat, related to Figure 3.

(**A**, **B**) Example place cells under LSD$_{high}$ (**A**) and LSD$_{low}$ (**B**), plotted same as in **Figure 2A**, **B**. Black arrows: dominant place field in PRE had similar firing locations with reduced rates in POST. Red arrows: place field became more bi-directional in POST.

(**C**) Four example place cells under the control condition. Two cells were from animals injected with saline in both PRE and POST (Saline). Two cells were from animals injected with saline in PRE, but the $5HT_{2A}R$ antagonist M100907 followed by LSD$_{low}$ (Ant + LSD$_{low}$) in POST.

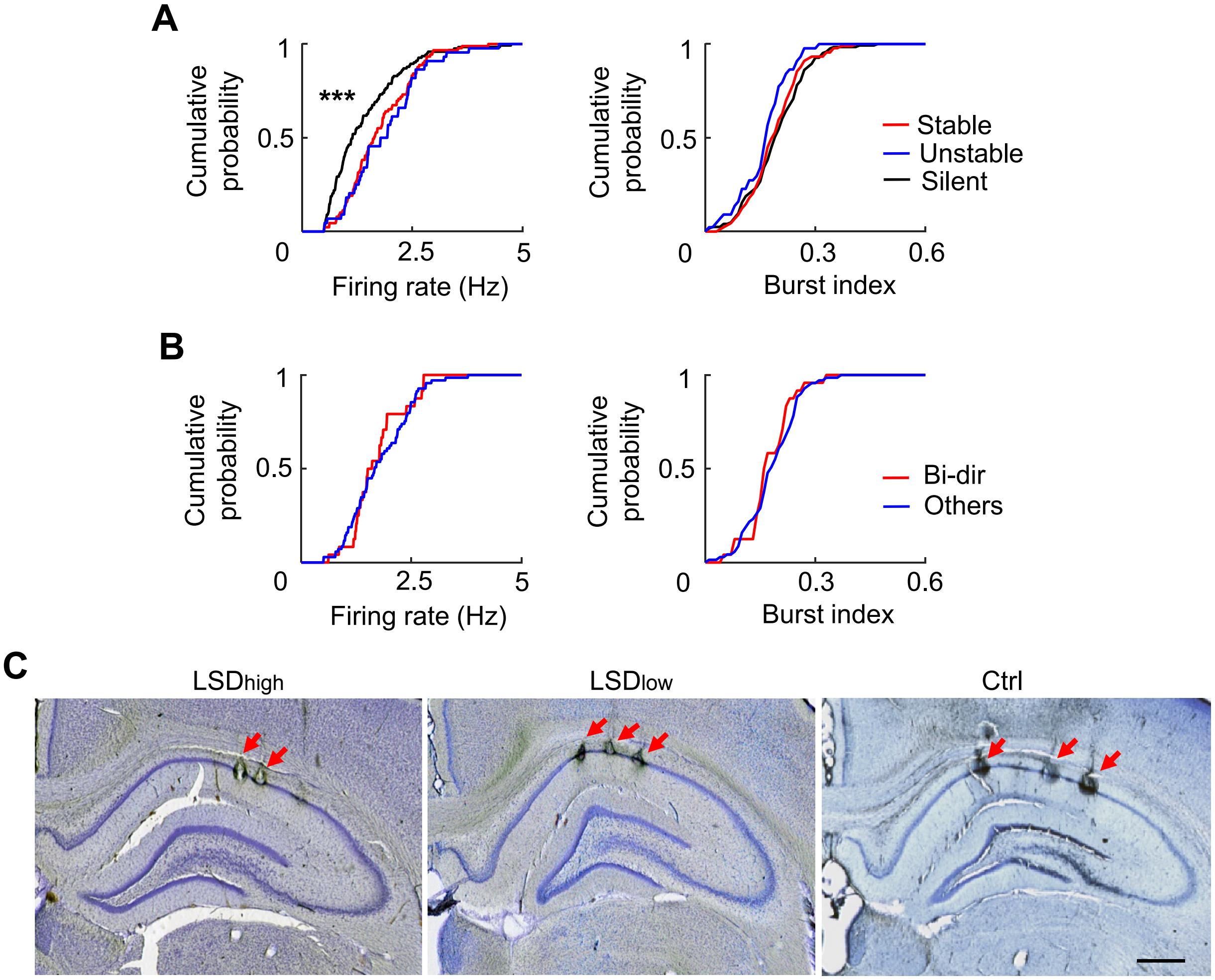

Figure S3. LSD appeared to reduce place cell rates broadly in the CA1, related to Figure 3.

(**A**) Cumulative distributions of firing rate and burst index in PRE for three groups of PRE-active cells under LSD (LSD_{high} and LSD_{low} combined): those with stable place fields in POST under LSD (stability between PRE and POST > 0.5; *N* = 89), those with unstable place fields (stability < 0.5; *N* = 44), and those silent in POST (rate < 0.5 Hz; *N* = 123). ****P* = 2.2 x 10^{-5}, *Kruskal-Wallis* test (post-hoc test after multiple-test correction between silent group and the other two groups: *P* < 1.8 x 10^{-4}). There were no obvious differences in burst index among the groups (*P* = 0.063). This result is consistent with the idea that LSD broadly reduced firing rates of CA1 cells such that the cells with low rates in PRE were more likely to go silent in POST.

(**B**) Same as (A), but for two groups of PRE-active cells: those becoming more bidirectional (Bi-dir: directional correlation POST-PRE > 0.2; *N* = 24) and other cells (Others: directional correlation POST-PRE < 0.2; *N* = 69). There were no differences in either firing rate (*P* = 0.50, *Mann-Whitney* test) or burst index (*P* = 0.83) between the two groups. The cells with increased bi-directionality in POST under LSD did not appear to form a special group.

(**C**) Tetrode recording sites (red arrows) in the CA1 of example animals under LSD_{high}, LSD_{low} and the control (Ctrl) condition. The tetrodes were implanted to the same targets in all animals under all conditions. Scale bar: 0.6 mm.

Supplemental Figure 4

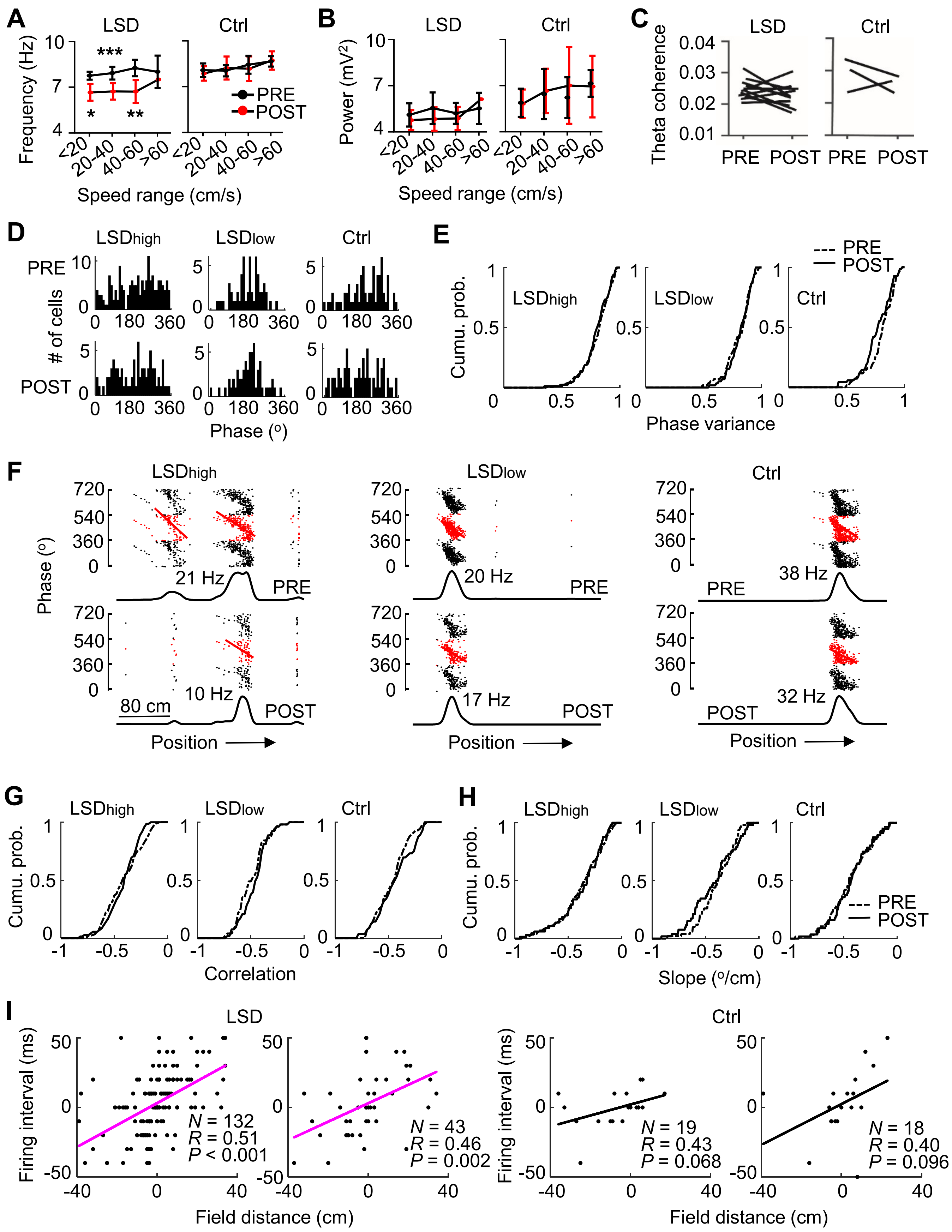

Figure S4. LSD affected theta oscillations modestly, but not theta phase properties or theta sequences of CA1 place cells, related to Figure 3.

(**A**, **B**) Average (mean ± SEM) CA1 theta peak frequency (**A**) and theta power (**B**) at different running speeds in PRE and POST under LSD (LSD_{high} and LSD_{low} combined; *N* = 13 sessions) and the control (Ctrl, *N* = 7) condition. Each dot is a session. Not all speed ranges were available for a session (1 LSD session available at the high speed of >60 cm/s; all other data points: *N* = 5 -1 sessions). The peak frequency significantly differed between PRE and POST under LSD (Mixed ANOVA, $P = 4.8 \times 10^{-7}$; **P* = 0.018, ***P* = 0.0022, ***$P = 2.4 \times 10^{-6}$, post-hoc *Welch's t*-test). There were no significant differences between PRE and POST in all other plots.

(**C**) Coherence between CA1 and VC LFPs averaged over the theta frequencies (6 - 10 Hz) during running in PRE and POST under LSD (*N* = 12 sessions) and the control (*N* = 3) condition. There were no significant differences between PRE and POST under LSD (*P* = 0.17*, Paired t*-test) or the control (*P* = 0.47*)* condition.

(**D**, **E**) Distributions of circular mean phase (**D**, shown as histograms) and phase variance (**E**, shown as cumulative distributions), which is between [0 1] and measures the degree of theta phase tuning, for CA1 active cells during running in PRE and POST under LSD_{high}, LSD_{low} and the control condition. There were no significant differences in either the mean phase (*P* > 0.40, *circular median* test) or the phase variance (*P* > 0.069, *Mann-Whitney* test).

(**F**) Theta phase precession (phase vs position) for example CA1 place fields in PRE and POST under LSD_{high}, LSD_{low} and the control condition, plotted in 3 theta cycles (y-axis). Each dot is a spike. Straight line: maximum linear regression between theta phases and field locations (red dots). Bottom curve: firing rate curve along the running (arrow) trajectory. Number: peak rate.

(**G**, **H**) Cumulative distributions of linear correlation (**G**) and regression slope (**H**) for CA1 place fields in PRE and POST under LSD_{high}, LSD_{low} and the control condition. There were no significant differences in either linear correlation (*P* > 0.19, *Mann-Whitney* test) or slope (*P* > 0.34) for all three connditions.

(**I**) Correlation between place field distances and firing time intervals within theta cycles, which was quantified by the peak time of pair-wise cross-correlation within theta cycles (within ±60 ms), for pairs of active CA1 cells with overlapping place fields in PRE and POST under LSD (LSD_{high} and LSD_{low} combined) and the control condition. Each dot is a pair. Line: linear regression. *R*, *P*, *N*: correlation, *P* value (*Pearson's r*) and number of pairs in each regression. The correlation here is used to quantify theta sequences of CA1 place cells with theta cycles. The number of pairs was relatively small, due to the requirement of overlapping place fields on the same trajectories. As a result, the regression was not statistically significant under Ctrl, however, its trend and the correlation value were comparable to LSD. Importantly, the correlation values under LSD in both PRE and POST were statistically significant and they were not different from each other (*P* = 0.38, *Fisher's exact* test).

Overall, theta oscillations appeared to be modestly impacted, but no obvious differences were found in theta phase tuning, phase precession or theta sequences for those cells that remained active during running in POST under LSD.

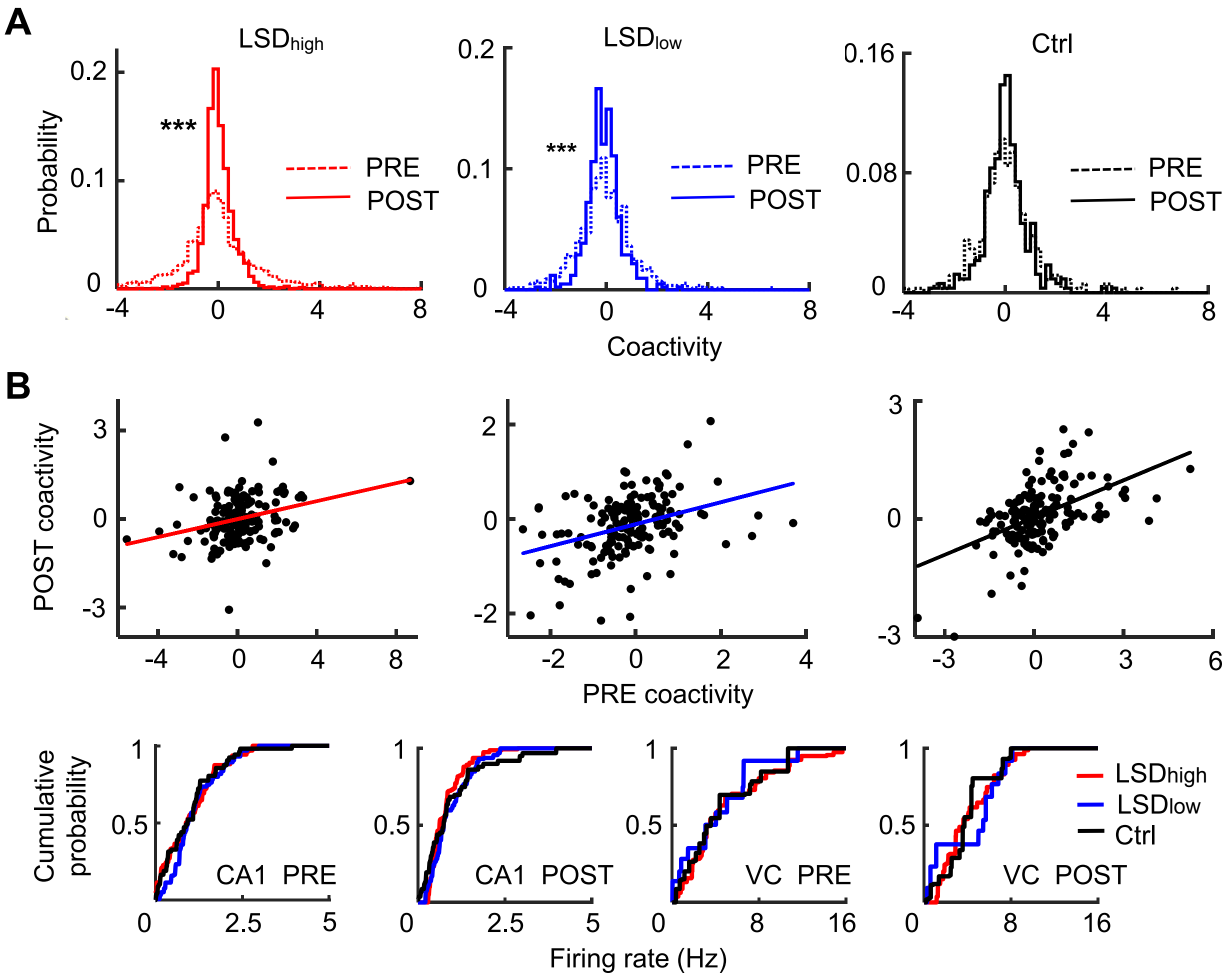

Figure S5. **Altered coactivity for CA1-VC active pairs during running under LSD, related to Figure 5.**

(**A**) Narrower distribution of coactivity under LSD. Each plot is the probability density function of coactivity in PRE and in POST for CA1-VC pairs under LSD_{high}, LSD_{low}, or the control (Ctrl) condition. There was a significant difference in the distribution between PRE and POST under LSD_{high} (PRE: N = 1910 cell pairs, POST: N = 1646, P = 3.9 x 10^{-35}, *Kolmogorov-Smirnov* test) and LSD_{low} (PRE: N = 550, POST: N = 415, P = 1.2 x 10^{-4}), but not the control (PRE: N = 342, POST: N = 324, P = 0.28) condition. *** P < 0.001.

(**B**) Reduced correlation between PRE and POST coactivity in downsampled CA1-VC pairs with same number of pairs and similar firing rates across conditions. Top: Coactivity values in PRE and POST for the downsampled pairs (LSDhigh: N = 159; LSDlow: N = 159; Ctrl: N = 159). The PRE-POST correlations in LSDhigh (R = 0.29) and LSDlow (R = 0.36) were significantly lower than that of Ctrl (R = 0.51; P = 0.010, 0.048 to LSDhigh, LSDlow respectively, *Fisher's exact* test). Bottom: cumulative distributions of firing rates under all 3 conditions in PRE and POST for the downsampled CA1 and VC cells. There were no significant differences in firing rate across the conditions in PRE/POST for CA1/VC cells (P > 0.14, *Kruskal-Wallis* test).

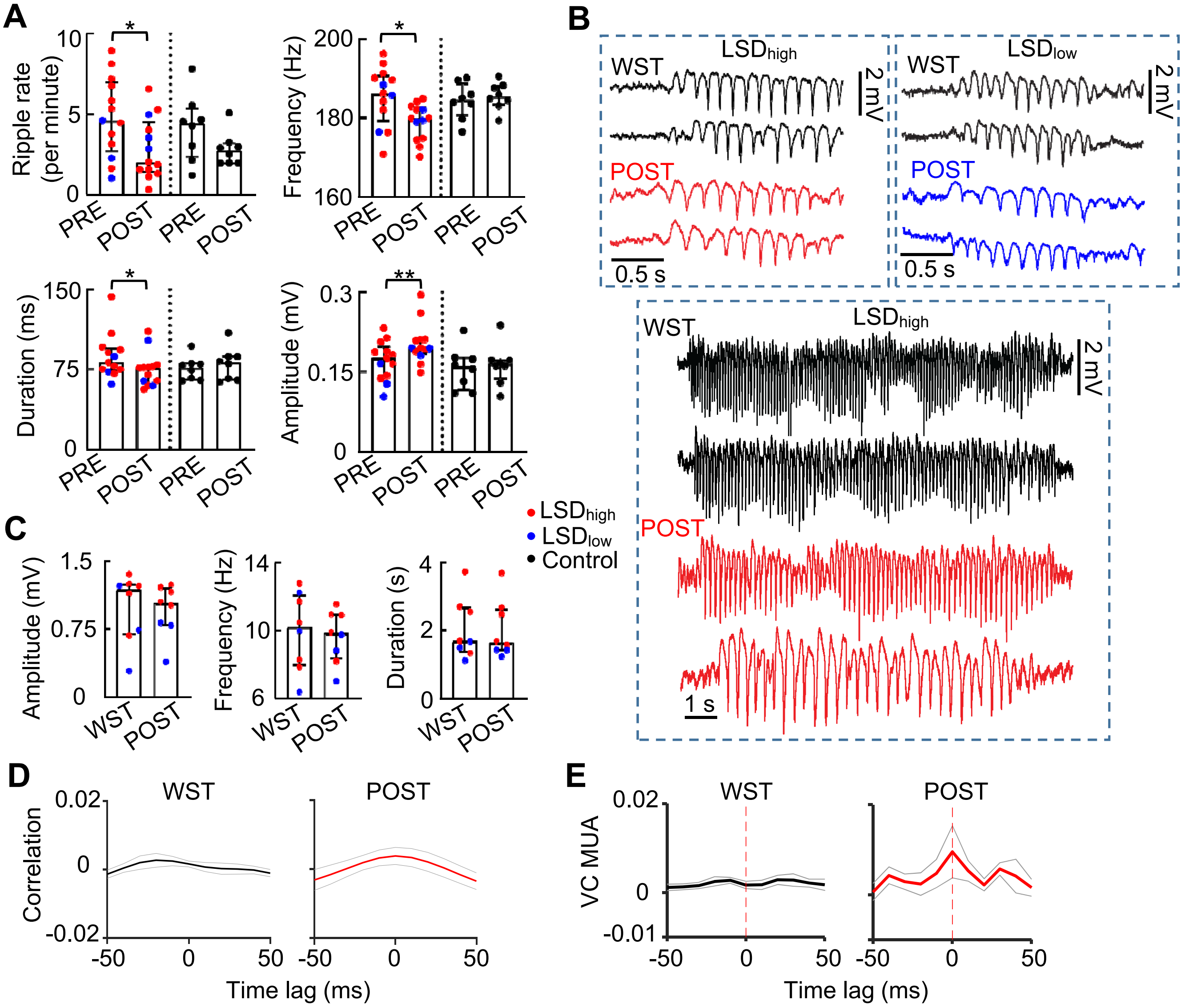

Figure S6. **Ripple and HVS characteristics in different sessions, related to Figure 6**.

(**A**) Ripple occurrence rate, frequency, duration, and amplitude in PRE and POST under LSD (LSD$_{high}$ and LSD$_{low}$ combined; N = 13 sessions) and the control (N = 8) condition. Each dot is a session in a separate rat. Box and bars: median and [25% 75%] range values. Ripples under LSD occurred less frequently in POST than in PRE and had slightly lower frequency, shorter duration and slightly higher amplitude. *P < 0.05, **P < 0.01, *Wilcoxon singed rank* test.

(**B**) Examples of HVS events in two animals under LSD$_{high}$ and one under LSD$_{low}$. In each box, two example VC LFP traces during WST in a sleep session (*top*) and two in POST on the track (*bottom*) are shown. HVS events in WST were detected in the SLEEP session after PRE, but before POST.

(**C**) Same as in (**A**), but for HVS amplitude, frequency, and duration in WST without LSD and in POST with LSD. There was no difference in any of the measures between WST and POST under LSD (P > 0.46, *Wilcoxon sign rank* test).

(**D**) Cross-correlation (mean ± SEM) between CA1 LFPs within ripple band and VC LFPs within HVS band in WST (N = 16 sessions) and in POST under LSD (N = 9). There was no significant peak in WST (*Two-way ANOVA*: P = 0.55) or in POST (P = 0.40). (**E**) Average (mean ± SEM) VC MUAs triggered by ripple peak times within ripples in WST (N = 11 sessions) and those in POST under LSD (N = 12). There was no significant peak in WST (*Two-way ANOVA*: P = 93) or in POST (P = 0.65). The results in **D&E** suggest no clear temporal relationship between CA1 ripples and cortical HVS.

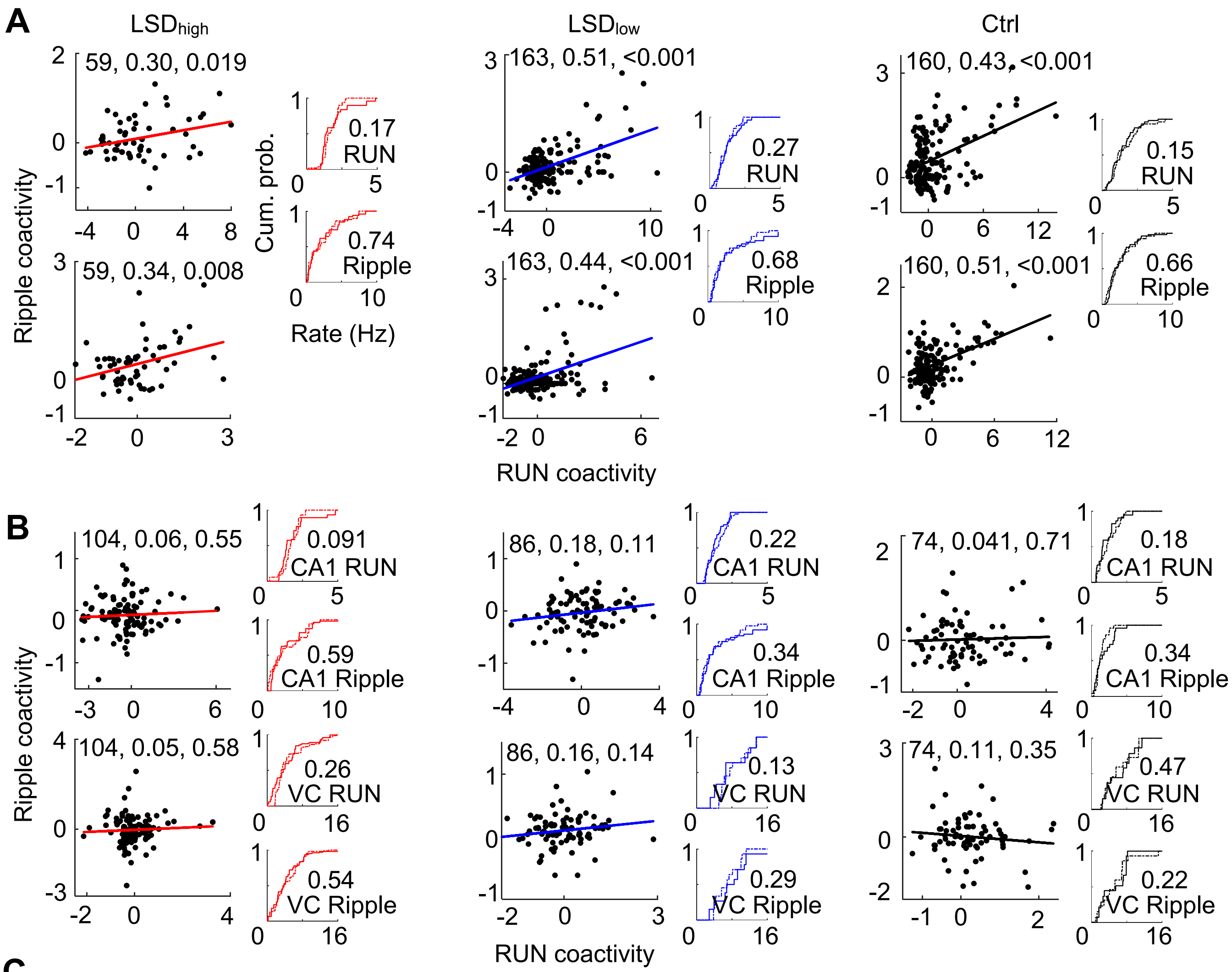

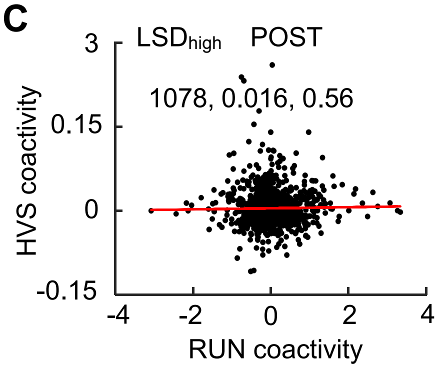

Figure S7. Correlating coactivity in running with coactivity within ripples in downsampled pairs, and with coactivity within HVS during immobility in CA1-VC pairs, related to Figure 7.

(**A**, **B**) Coactivity during running (RUN) and within ripples for downsampled CA1 pairs (**A**) and for CA1-VC pairs (**B**) in PRE/POST under LSD_{high}, LSD_{low}, and the control (Ctrl) condition. Each dot is a pair. Solid line: linear regression. Numbers: # of pairs, *Pearson's r,* and associated *P* value in each regression. Insets: cumulative distribution of firing rates of downsampled CA1 or VC cells. *Number*: *P* value comparing PRE and POST (*Wilcoxon signed rank* test).

(**C**) Coactivities during running and within HVS events for CA1 -VC cell pairs. The coactivity was computed within HVS events in POST under LSD_{high}, since total HVS event duration was short in PRE and under other conditions. Each dot is a pair. Note the non-significant correlation between RUN and HVS coactivities, suggesting the lack of awake reactivation across CA1 and VC within HVS events.
